# Understanding the influence of design-related factors on human–AI teaming in a face matching task

**DOI:** 10.1186/s41235-025-00701-x

**Published:** 2026-01-07

**Authors:** Eesha Kokje, Eva Lermer, Christopher Donkin, Susanne Gaube

**Affiliations:** 1 Center for Leadership and People Management, Department of Psychology, LMU Munich, Giselastr. 10, 80802 Munich, Germany; 2 Department of Business Psychology, Technical University of Applied Sciences Augsburg, Augsburg, Germany; 3 Department of Psychology, LMU Munich, Munich, Germany; 4 Global Business School for Health, UCL, London, UK

**Keywords:** Artificial intelligence, Human–AI interaction, Face matching, AI advice, Decision-making

## Abstract

There has been a steep rise in the use of decision aids enabled by artificial intelligence (AI) for facial identity verification. The use of such systems and the impact of their implementation on human decision-making is still not well understood. The current study aimed to explore factors associated with the design of the paradigm and of the presentation of AI-enabled predictions. Across three pre-registered experiments, we examined the impact of (a) stated AI accuracy, (b) mismatch frequency (i.e. proportion of match and mismatch pairs), and (c) advice type (binary only vs. binary + similarity rating) on performance in a one-to-one face matching task. Participants’ performance generally improved when aided by AI compared to a baseline without decision support. The largest improvement was observed when no information on the AI’s overall accuracy was provided. Further, the frequency of mismatches did not influence performance, but biased responses. Finally, similarity ratings marginally improved overall performance and increased users’ certainty in their decisions, but did not help to dismiss inaccurate predictions. Additionally, two findings were consistent across all experiments. First, participants often failed to dismiss inaccurate AI predictions, resulting in significantly lower performance compared to accurate predictions. Second, on a group level, the human–AI team did not outperform the AI alone, though examination of individual performance showed that some participants were able to exceed the AI’s accuracy. These findings contribute towards determining appropriate design formats for AI prediction in a human-in-the-loop system, so that the performance of the human–AI team can be maximised.

**Supplementary Information:**

The online version contains supplementary material available at 10.1186/s41235-025-00701-x.

## Introduction

Decision-making aids enabled by artificial intelligence (AI) are becoming increasingly prevalent in a number of different fields (Coombs et al., 2020). Applications range from human resources management, such as CV screening software, to finance applications like fraud detection or credit scoring, and healthcare uses like clinical decision support systems for diagnosis and treatment selection (Albert, 2019; Aziz & Andriansyah, 2023; Hudecek et al., 2024; Rajpurkar et al., 2022). In most, especially high-stakes cases, these decision aids are not equipped to operate independently but are used in tandem with humans (Gupta et al., 2022; Kulju et al., 2019), in what is known as a *human-in-the-loop* system. This gives rise to several challenges in the effective deployment and integration of AI-enabled decision aids within existing systems, as successful cooperation largely depends on the users’ effective uptake of advice from the AI systems.

One major issue arising from humans having to interact with AI aids when making decisions is finding the right balance between trusting the algorithm and using one’s own judgement. On the one hand, some studies show that there is a distrust of and reluctance to accept AI advice among users i.e. *algorithmic aversion* (Jussupow et al., 2020). For example, individuals who view themselves as task experts in a domain may resist relying on AI predictions due to an overconfidence in their own ability above that of the AI (Bayer et al., 2022; Logg et al., 2019). Other studies have shown a preference for algorithmic advice over advice from other humans, i.e. *algorithmic appreciation* (Logg et al., 2019), such as when the task is perceived to be difficult (Bogert et al., 2021). When users are unable to balance their trust with scepticism in the algorithm and accept inaccurate AI advice, *overreliance* occurs (Kostick-Quenet & Gerke, 2022; Schreck et al., 2023). With the increasing use of AI-enabled decision aids, it is important to identify and solve the potential issues of algorithm aversion and overreliance, and design optimal presentation formats for AI predictions to facilitate the effective and safe implementation of the technology. This paper employs a one-to-one face matching paradigm to examine the factors potentially influencing effective interaction of humans with AI aids.

### Artificial intelligence in face matching

Person identity verification via faces is commonly employed in day-to-day life, such as in identity checks via photo IDs (Towler et al., 2017). Despite performing this task on a regular basis, the performance of humans on one-to-one face matching tasks is known to be suboptimal. Error rates between 10 and 20% are commonly reported when conditions are ideal, even among individuals performing the task professionally (Burton et al., 2010; White et al., 2014). Performance drops further under more unfavourable conditions like reviewing images with lower quality, older images, images of a person from another race, and under time pressure (Fysh & Bindemann, 2017). It is becoming increasingly common to use automatic face matching software for this task, particularly in high-stakes areas such as border control (Lehtonen & Aalto, 2017). While top-performing AI systems outperform most humans on high-quality images, other systems are not as good; and even the top-performing systems struggle with images of low-quality or bad illumination (Haq et al., 2025; Merino et al., 2025). Thus, human involvement is required to spot errors or when results are inconclusive (Carragher & Hancock, 2020; Grother et al., 2021; Phillips et al., 2018). Moreover, they are not enabled to operate completely independently, at least as of now, and require human oversight, according to the current European Border Agency guidelines (FRONTEX, 2015).

Individually, both humans and AI aids are susceptible to errors, but together they may achieve higher performance, as each may be capable of overcoming the shortcomings of the other (Inkpen et al., 2022; Jarrahi, 2018; Johnson & Vera, 2019; Kamar, 2016). If, however, the AI aids are implemented suboptimally, this optimistic vision of human–AI collaboration might not be achieved. Therefore, it is important to study the conditions that maximise performance, as even low error rates in high-stakes conditions can have major consequences. Taking the example of identity verification, considering the number of people that go through border control every day, even a 2% error rate would be a large number. As an example, London’s Heathrow Airport, one of the busiest in the world, processed over 7.3 million international travellers in July 2023, which is over 235,000 passengers daily (https://mediacentre.heathrow.com/pressrelease/detail/17399). A 2% error rate, in this case, is the equivalent of 4700 falsely identified passengers. In sensitive cases, such as an instance of human trafficking or a criminal crossing the border, even a single error can have drastic consequences.

So far, there have been only a few studies investigating human–AI interaction in the context of a face matching task. Howard et al. (2020) examined the effect of the implied source of advice and found that participants trusted their own judgement the most, followed by advice from AI. The judgement of other humans was rated as least trustworthy. Interestingly, the source of advice did not impact participants’ performance. Carragher et al. (2024), on the contrary, reported a positive impact of higher trust. Participants with a generally higher tendency for trust in automation, and those who trusted AI’s decisions more than another human’s judgement in a face matching task, were seen to benefit more performance-wise when aided by AI.

Fysh and Bindemann (2018) investigated the effect of instructions, AI accuracy, and performance feedback. A key finding was that irrespective of whether participants were instructed to ignore or attend to the AI prediction, accuracy was higher when accurate AI predictions were presented. The authors concluded that participants factored the AI prediction into their decision, even when specifically instructed to ignore it. Further, following a round in which only accurate predictions were shown, and participants received feedback on their performance, they relied increasingly on the AI prediction resulting in a greater number of errors when encountering inaccurate predictions afterwards. This indicates that a positive experience with the AI can result in an increase in trust, but also in an increase in overreliance on inaccurate predictions.

In another study (Carragher & Hancock, 2023), two AI systems with drastically different accuracy rates were tested. Participants tended to place greater reliance on the predictions of the more accurate AI, even when they were not informed of the AI’s accuracy levels. This finding suggests that users are able to judge the AI’s accuracy to a certain extent, but not fully so, since participants performed significantly worse when inaccurate advice was presented. Further, they also examined the impact of presenting similarity ratings for face pairs in addition to binary match/mismatch decisions. Another study (Mueller et al., 2024) also examined the impact of providing similarity ratings instead of binary predictions. Both studies did not find an advantage of presenting similarity ratings over binary predictions in terms of performance, but Mueller et al. found that responses were more biased with binary predictions.

In an ideal scenario, using AI-generated predictions to support human decisions should result in overall better performance, since combining AI and human abilities might help overcome the shortcomings of each party (Towler et al., 2023). However, the findings from the above studies do not support this. Humans, aided by AI systems, did not surpass AI accuracy in any of the studies, suggesting that design and implementation strategies need to be carefully examined (Amershi et al., 2019). Therefore, the goal of this study was to investigate design factors of the paradigm itself and the AI predictions that could potentially influence the effective deployment of AI systems in practice to enhance performance. In a series of pre-registered experiments, we manipulated aspects of the paradigm design, prediction design, and decision context to find the most appropriate formats that would enhance the overall collaborative performance.

## General method

All three experiments consisted of a one-to-one face matching task, wherein two human faces were presented simultaneously on a screen. The two faces either belonged to the same person (match) or to different people (mismatch). Participants were tasked with making match/mismatch judgements for each face pair. Additionally, with the exception of the baseline condition, a prediction from an AI model was presented to help them decide. The study received ethical approval from the University Hospital Regensburg. The experiments were pre-registered on OSF (Experiment 1, Experiment 2, Experiment 3). A power analysis was conducted in MorePower 6.0.4 (Campbell & Thompson, 2012) prior to Experiment 1 assuming a medium to large effect size (*η*^2^ = 0.1) for a 2 × 4 within-subject paradigm (as a suitable prior effect size could not be identified), with 80% power and α = 0.05, which indicated a sample size of 74. We aimed to recruit ~ 100 participants to account for losses due to exclusions and non-completion. We aimed for the same sample size for subsequent experiments.

### Stimuli

**Fig. 1:**
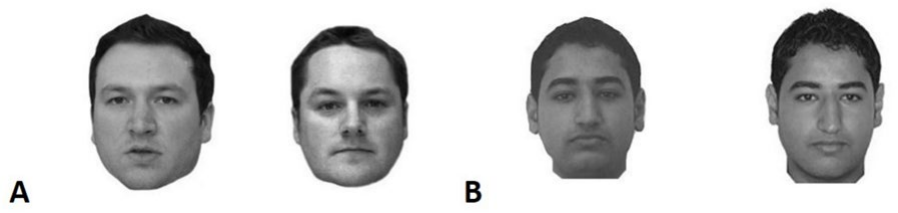
An example of the stimuli used in the experiments. The example depicts a (**A**) mismatch pair from the GUFD and a (**B**) match pair from the Arab face database

The stimuli consist of face pairs in full-face frontal view, presented in greyscale on a white background, with a neutral expression, and all external background information removed. This means that photos were closely cropped so that no external cues such as clothing, accessories, or background were visible, and only the head and face were visible (see Fig. 1). A total of 200 face pairs were included. Of these, 100 pairs were taken from the Glasgow University Face Database (GUFD; Burton et al., 2010), containing images only of Caucasian faces, and 100 from an Arab database, containing images only of Arab faces (Megreya & Burton, 2008). All face pairs were male, as female faces for the Arab database were not available. We wanted to introduce diversity in the stimuli, and took into account that, in humans, the other-race effect is a more robust effect compared to gender-based effects (Fysh & Bindemann, 2017), and thus, ultimately chose to introduce ethnic diversity. Half of the face pairs in each database depicted the same person (match), and half depicted different people (mismatch). The detailed methods for the construction of the face arrays are available in the original studies (Burton et al., 2010; Megreya & Burton, 2008).

### AI predictions

An openly available deep convolutional neural network (DCNN) AI model called Facenet (Schroff et al., 2015), within deepface (https://github.com/serengil/deepface), a face recognition and facial attribute analysis framework for Python, was used to generate the predictions, which were later used in the experiments. The model processed 200 pairs, 100 from each database (GUFD and the Arab face set). The model provides a ‘distance’ value, i.e. the degree of dissimilarity between the two faces, which ranges from 0 to 1, with higher values indicating greater distance. A binary prediction of ‘match’ or ‘mismatch’ is produced based on the distance value. Facenet uses a threshold value of 0.40, and all pairs with a ‘distance’ > 0.40 are classified as mismatches. The model was found to be 92.9% accurate. Of the 14 cases where errors were made, 8 were match cases (7 from the Arab database and 1 from the Glasgow database), and 6 were mismatch cases (4 from the Arab database and 2 from the Glasgow database). For two cases, the algorithm returned an error to classify instead of a prediction, and thus these could not be successfully classified, which we then excluded. The cases were not replaced with alternate cases. We attempted to maintain the accuracy rate close to 92.9% across all experiments, in order to mimic real conditions.

### Procedure

The experiment was programmed in PsychoPy (Peirce, 2007). Participants provided informed consent and completed the experiment online starting with a short demographic questionnaire on Qualtrics, followed by the experiment completed on Pavlovia. Each trial began with a fixation cross for one second, followed by the presentation of the face pair along with the AI prediction (when applicable), and ended when a response was registered. Participants’ responses on each trial were binarily coded as correct (hits & true negatives for match and mismatch stimuli, respectively) or incorrect (misses & false positives), and their overall performance was calculated as the percentage of correct decisions. The trials were grouped and organised into blocks, with each block containing an equal number of trials. The trial order within each block was randomised for each participant. At the end of each block, participants were asked to rate the ‘confidence in your decisions’, ‘task difficulty’, and ‘AI usefulness’ (when applicable), on a scale from 1 to 10, with 1 being ‘not at all…’ and 10 being ‘very…..’. Participants were encouraged to take a short break after the completion of each block.

### Analyses

We conducted mixed-effects logistic regressions for performance, with participants and face pairs as random factors. We specify the fixed factors for each analysis in the respective ‘Results’ section. For significant effects, we followed up with pairwise comparisons of estimated marginal means (EMM) using Holm correction for multiple testing. For AI usefulness, confidence, and difficulty rating, we conducted linear mixed-effects regressions with the condition as the fixed factor and participants as a random factor. Additionally, we also analysed the data within the Signal Detection Theory (SDT) framework. The details of the analysis and the results are in the supplementary material.

## Experiment 1

One issue that repeatedly comes up in the implementation of AI tools is that of transparency, and the argument that greater transparency increases trust in AI, which should lead to better outcomes (Andrada et al., 2023; Zerilli et al., 2022). One aspect of transparency is meta-information about the algorithm, and in particular, the overall accuracy of the algorithm. AI developers leverage the accuracy rates of their algorithms as a selling point (Grother et al., 2019). However, the effect of revealing the AI’s accuracy to users, on their performance, has been mixed (Carragher & Hancock, 2023; Eisbach et al., 2023; He et al., 2023). In the current study, we were interested in users’ *perception of an algorithm’s accuracy* to understand whether they adjust their reliance behaviour according to their perception of the algorithm’s capability. Previous studies have shown that users fail to overrule inaccurate AI predictions, and a lower algorithm accuracy rate leads to poorer performance (Carragher & Hancock, 2023; Matzen et al., 2020). In order to avoid performance being affected by the actual number of incorrect predictions and considering that we were interested in *perception*, we only manipulated the *stated accuracy rate* of the algorithm. What we mean by stated accuracy rate is that participants were made to believe that three different algorithms with varying accuracy rates were used, and the fictitious rates were presented. In reality, the same algorithm, detailed above, was used to generate the predictions in all conditions, and the *true accuracy rate* was the same across the conditions. In addition, there was a control condition wherein no AI prediction was presented.

The aims of the experiment were: first, to establish the baseline performance^1^ without AI and evaluate whether it improves when an AI prediction is presented. Second, to examine whether stated accuracy rate influences participants’ performance, i.e. whether users adjust their abidance with AI prediction, in accordance with their perception of the accuracy of the AI tool. We were also interested in how stated accuracy influenced abidance behaviour for accurate and inaccurate predictions, respectively.

### Participants

The participants were recruited from universities (LMU Munich, University of Regensburg, and FOM Hochschule) and received course credit in exchange for their participation. A total of 99 individuals completed the study. We excluded three participants with an overall performance below 50%, as this falls below chance level, one who had an average response time < 1 s, and one fulfilling both of these criteria. Since the study was conducted online, we were highly cautious, as both of these could indicate a lack of attention. Ninety-four participants were included in the analysis. Demographic characteristics of our sample from Experiment 1 are presented in Table 1.

**Table 1:** Demographic characteristics of participants in Experiment 1 (*1 participant did not complete the questionnaire)

	Overall
	(*N* = 94)
Age	
Mean (SD)	23.2 (4.31)*
Median [Min, Max]	22.0 [18.0, 42.0]*
Gender	
Female	61 (64.9%)
Male	31 (33.0%)
Non-binary	1 (1.06%)
Unknown*	1 (1.06%)
Country	
Germany	92 (97.9%)
Other	1 (1.06%)
Unknown*	1 (1.06%)
Ethnicity	
East Asian	2 (2.13%)
Middle Eastern/Arab	2 (2.13%)
Other/Mixed	5 (5.32%)
South Asian	1 (1.06%)
White/Caucasian	83 (88.3%)
Unknown*	1 (1.06%)

### Stimuli and procedure

In this experiment, the stimuli, as described in the ‘General Method’, were paired with four different AI prediction conditions. One was a *control condition*, wherein no AI prediction was presented. In the three *experimental conditions*, the actual predictions from the Facenet algorithm were presented and paired with three different stated accuracy rates: 95% (high), 70% (low), and unknown.

A total of 192 face pairs were presented, which were equally distributed across four blocks consisting of 48 pairs in each. Of the four blocks, two blocks contained three trials and two blocks contained four trials with inaccurate AI predictions (Block 1: 2 match, 1 mismatch; Block 2: 1 match, 2 mismatch; Block 3: 2 match, 2 mismatch; Block 4: 3 match, 1 mismatch). Pairing each block with each of the four conditions, which was counterbalanced among participants, ensured that none of the conditions was adversely affected by the unequal distribution of the inaccurate predictions. An average accuracy rate of 92.7% was maintained across the four blocks.

**Fig. 2:**
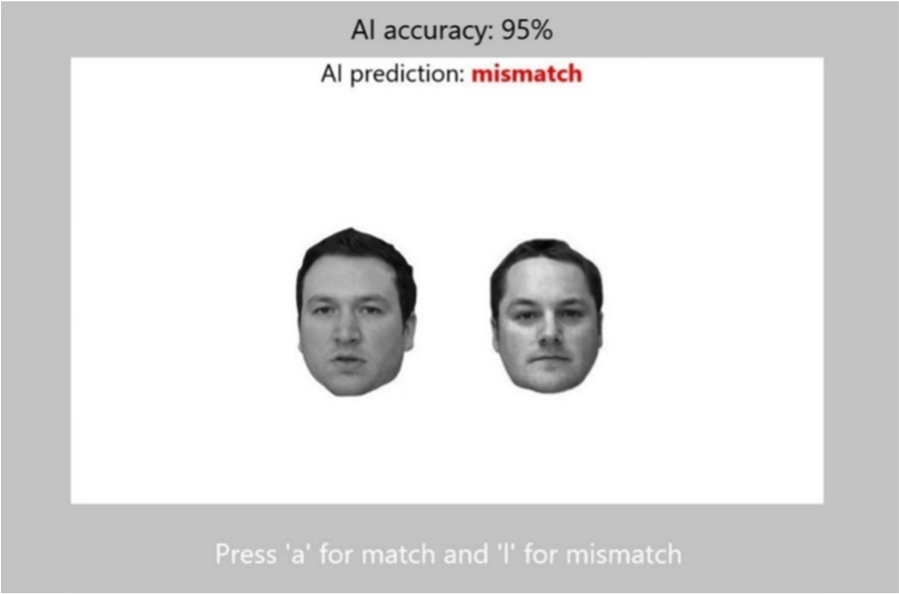
An example of the information presented on screen on each trial. The value of AI accuracy presented changed according to the condition. The AI prediction was presented as a match (in green) or mismatch (in red)

Each block was assigned to one of the four conditions. At the beginning of each block, participants were informed of the condition or (fictitious) algorithm being presented in that block (e.g. Block 1—AI accuracy rate: 95%), and this information stayed on screen throughout the block. Participants were also explicitly informed before the start of the experiment that the accuracy rate displayed “represents the overall accuracy level of the AI system (i.e. percentage of correct matching decisions overall)”, to avoid misunderstandings about what the number may represent. Participants made the matching decision with a key press—‘a’ for match and ‘l’ for mismatch. This information was also presented at the bottom of the screen on each trial to remind participants. An example of how the information was presented on each trial is shown in Fig. 2.

Four versions of the experiment were created for the purpose of counterbalancing, wherein each block of stimuli was paired with each of the four conditions. Each participant only completed one version. The order of presentation of the four conditions was also counterbalanced across participants so that the order of presentation did not bias their responses.

### Results and discussion

A 4 (stated accuracy rate: control vs. 95 vs. 70% vs. unknown) × 2 (AI prediction accuracy: accurate vs. inaccurate) within-subject design was employed.

#### Performance

**Fig. 3:**
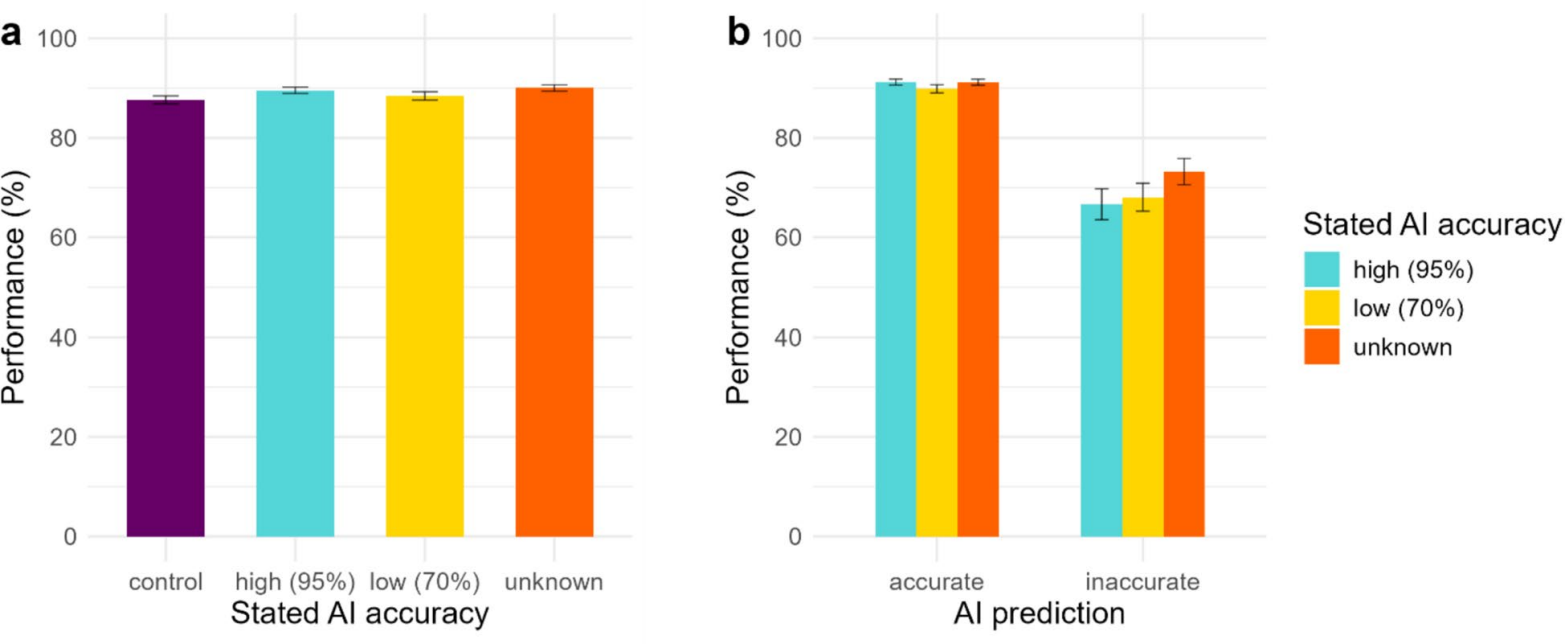
Overall performance (**a**) in each of the four conditions, and (**b**) in the three experimental conditions for accurate and inaccurate AI predictions. Error bars represent standard error

An overview of the mean performance in each of the four conditions is presented in Fig. 3. Performance was highest for the unknown condition and lowest for the control condition. A mixed-effects logistic regression with stated accuracy rate as a predictor, and participants and face pairs as random factors, was conducted for performance. This is a deviation from our pre-registration which specified within-subject ANOVAs. This deviation is for the purpose of maintaining consistency across all the experiments in this paper (see supplementary material for ANOVA results).

The results show that participants performed significantly better in all three conditions with AI predictions compared to the control condition (Table 2). However, post hoc pairwise comparisons revealed that only the high and unknown conditions were significantly different from the control condition (both *p*s < 0.001), but the low condition was not (*p* = 0.056). Other comparisons, high vs. low (*p* = 0.226), high vs. unknown (*p* = 0.838), and low vs. unknown (*p* = 0.224) were non-significant. As an additional analysis, we examined how many participants exceeded the AI’s accuracy. The highest number was in the unknown condition (40), followed by low accuracy (31), and high accuracy and control conditions (30 each).

**Table 2:** Results of the mixed-effects logistic regression (Experiment 1)

Predictors	Performance (Model 1)					Performance (Model 2)				
	Odds ratios	SE	95% CI	Statistic	*p*	Odds Ratios	SE	95% CI	Statistic	*p*
Intercept	11.78	1.40	9.34–14.86	20.82	** < 0.001**					
Condition [95%]	1.34	0.10	1.16–1.55	4.04	** < 0.001**					
Condition [70%]	1.19	0.09	1.04–1.37	2.46	**0.014**					
Condition [unknown]	1.36	0.10	1.18–1.58	4.21	** < 0.001**					
Intercept						19.00	2.37	14.88–24.27	23.60	** < 0.001**
Condition [70%]						0.83	0.07	0.71–0.97	− 2.29	**0.022**
Condition [unknown]						0.96	0.08	0.81–1.12	− 0.55	0.579
AI prediction [inaccurate]						0.14	0.05	0.07–0.28	− 5.44	** < 0.001**
Condition [70%] x AI prediction [inaccurate]						1.74	0.41	1.09–2.76	2.34	**0.019**
Condition [unknown] x AI prediction [inaccurate]						1.61	0.38	1.01–2.56	2.01	**0.045**

Model 1: stated accuracy rate; Model 2: stated accuracy rate x AI prediction accuracy

**Model 1**: Random effects: *σ*^2^ = 3.29, τ_00 TrialID_ = 1.42, τ_00 participant_ = 0.34, ICC = 0.35,* N*_TrialID_ = 192,* N*_participant_ = 94, observations = 18,048, marginal *R*^2^ = 0.003/conditional *R*^2^ = 0.351. **Model 2:** Random effects: σ^2^ = 3.29, τ_00 TrialID_ = 1.33, τ_00 participant_ = 0.34, ICC = 0.34, N_TrialID_ = 192, N_participant_ = 94, observations = 13,536, marginal *R*^2^ = 0.036/conditional R^2^ = 0.361. OR > 1 is associated with higher odds for correct decision; OR < 1 is associated with lower odds for correct decision. The respective intercepts indicate that the probability of a correct decision was 0.92, & 0.95 in models 1 & 2, respectively. Including ‘stated AI accuracy condition’ improved to the model fit of Model 1 over the null model, (χ^2^(3) = 22.16, *p* < 0.001) and including ‘implied AI accuracy condition’ and ‘AI prediction accuracy’ improved model fit of Model 2 over the null model and the model with ‘stated AI accuracy condition’ only (χ^2^(1) = 22.12, *p* < 0.001)

Next, only data from the three blocks where the AI prediction was presented were included in the following analyses. A mixed-effects logistic regression was conducted, with stated accuracy rate, AI prediction accuracy and their interaction as fixed effects, and participants and face pairs as random factors. The results showed a significant effect of AI prediction accuracy with participants performing 19.3% better when accurate predictions were presented (Fig. 3). This is also reflected in the increased sensitivity on trials with accurate predictions (see supplementary materials for SDT analysis). It is also worth noting that performance in the inaccurate AI prediction condition was worse than the baseline performance. A significant interaction effect was also observed. However, pairwise comparisons did not show significant contrasts between the stated accuracy rate levels for accurate: high vs. low (*p* = 0.066), high vs. unknown (*p* = 0.580), low vs. unknown (*p* = 0.168), or inaccurate predictions: high vs. low (*p* = 0.191), high vs. unknown (*p* = 0.158), low vs. unknown (*p* = 0.796). It is important to note here that there were only 14 face pairs with inaccurate advice compared to 178 face pairs with accurate advice. Results for AI usefulness, confidence, and difficulty are reported in the supplementary material.

Although the highest mean performance was observed in the unknown condition and the lowest in the low stated accuracy rate condition, indicating that not revealing the accuracy rate of the algorithm may be favourable, it is important to note that the performances were not significantly different. Interestingly, even in the condition where participants’ performance was highest, their average performance as a group did not exceed that of the AI alone. The lack of a ubiquitous positive effect of collaboration indicates that further studies are required to examine other aspects of design of human–AI collaboration systems.

## Experiment 2

In face matching experiments, the design is typically 50–50% ratio of match and mismatch identities. However, in real-life situations requiring one-to-one face matching, the frequency of mismatches tends to be much lower. For example, in a border control scenario, cases where false photo ID documents are presented are rare (Bindemann et al., 2010). It is possible that in scenarios where a *match* is the norm, detecting *mismatches* may be more challenging compared to more balanced scenarios. Previous studies investigating the effect of mismatch frequency in face matching tasks have reported mixed results. Two studies (Baker & Bindemann, 2025; Bindemann et al., 2010) did not report significant differences in performance depending on mismatch frequency, whereas Papesh and Goldinger (2014) found that participants were significantly worse at detecting mismatches when they occurred infrequently. Additionally, several studies (Baker & Bindemann, 2025; Papesh & Goldinger, 2014; Stabile et al., 2024) reported that participants shifted their criterion in response to low mismatch frequencies to optimise their responses for the higher base rate of matches.

In addition to these mixed results, it is also unclear what effect mismatch frequency has on performance in a scenario involving AI decision aids. It is unknown whether or not users’ patterns of abidance with the AI prediction differ depending on the frequency of mismatches encountered. For example, users may anticipate more or fewer mismatches and reject AI predictions more frequently, if the ratio deviates from their expectations, assuming the predictions must be inaccurate. A study by Mueller et al. (2024), which used a low mismatch frequency design (10% mismatches), reported that participants were biased to responding with a match, especially when only binary match/mismatch predictions were provided. This study, however, did not make a direct comparison of low and balanced mismatch frequency, and previous studies have shown that people generally have lower accuracy for mismatch trials compared to match trials (Burton et al., 2010; Kokje et al., 2018; Tummon et al., 2019). So, it is unclear whether the lower accuracy on mismatch trials resulted from a response bias.

Our experiment aimed to evaluate the effect of mismatch frequency on face matching performance in a human–AI interaction scenario by making a direct comparison of low and balanced mismatch frequency scenarios. We compared three mismatch frequency conditions and their interaction with the AI prediction accuracy. Unlike Mueller et al., we did not inform participants about the mismatch frequency rates. Since we were interested in specifically assessing the detection of mismatches as a result of mismatch frequency, we also examined performance separately according to trial type—match or mismatch (i.e. face pairs which were match cases and those which were mismatch cases).

### Participants

Participants were recruited from Prolific and received monetary compensation for their participation. Ninety participants completed the study. Outliers (based on boxplots), participants performing below chance (< 50% performance), or those finishing unrealistically quickly (average response time < 1 s) were excluded. This resulted in 78 participants being included in the analysis. Table 3 displays the demographic characteristics of the sample from Experiment 2.

**Table 3:** Demographic characteristics of participants in Experiment 2

	Overall
	(*N* = 78)
Age	
Mean (SD)	27.5 (7.72)
Median [Min, Max]	25.5 [18.0, 50.0]
Gender	
Female	30 (38.5%)
Male	48 (61.5%)
Country	
Germany	78 (100%)
Ethnicity	
White/Caucasian	78 (100%)

### Stimuli and procedure

In this experiment, we manipulated the frequency of mismatch trials across three blocks, each containing 40 trials. One block, the *equal mismatch* condition had an equal ratio (50%) of match and mismatch trials. A second block, which was the *low mismatch* condition, had 90% match trials and 10% mismatch trials. A third block, the *very low mismatch* condition, had 95% matches and 5% mismatches. All participants completed all three blocks. The order of the blocks was counterbalanced among participants. Participants received no information regarding the mismatch frequency; only that they would encounter some match and some mismatch trials. The *low mismatch* condition contained only two more mismatch trials compared to the *very low* condition, a difference participants are unlikely to notice. In the real world, individuals would not have information about the exact mismatch frequency, and this frequency would may also very slightly depending on settings or on a day-to-day basis in the same setting. We wanted to examine whether these minor differences would influence performance. Participants also did not receive any information regarding the accuracy rate of the AI. Within each block, three trials were paired with inaccurate advice (*equal*: 1 match, 2 mismatch; *low*: 2 match, 1 mismatch; *very low*: 2 match, 1 mismatch). We attempted to balance the number of incorrect predictions for matches and mismatches. Due to the limited number of mismatch cases in the low and very low conditions, we decided to only have 1 case of incorrect prediction in each condition for mismatches. To then somewhat balance this out, we decided to reverse the proportion in the equal condition. Within blocks, the order of face pairs was completely randomised. Participants registered their decision with a key press—‘a’ for match and ‘l’ for mismatch.

### Results and discussion

A 3 (mismatch frequency: *equal* vs *low* vs *very low*) × 2 (trial type: *match* vs *mismatch*) × 2 (AI prediction accuracy: *accurate* vs *inaccurate*) within-subject design was employed.

#### Performance

**Fig. 4:**
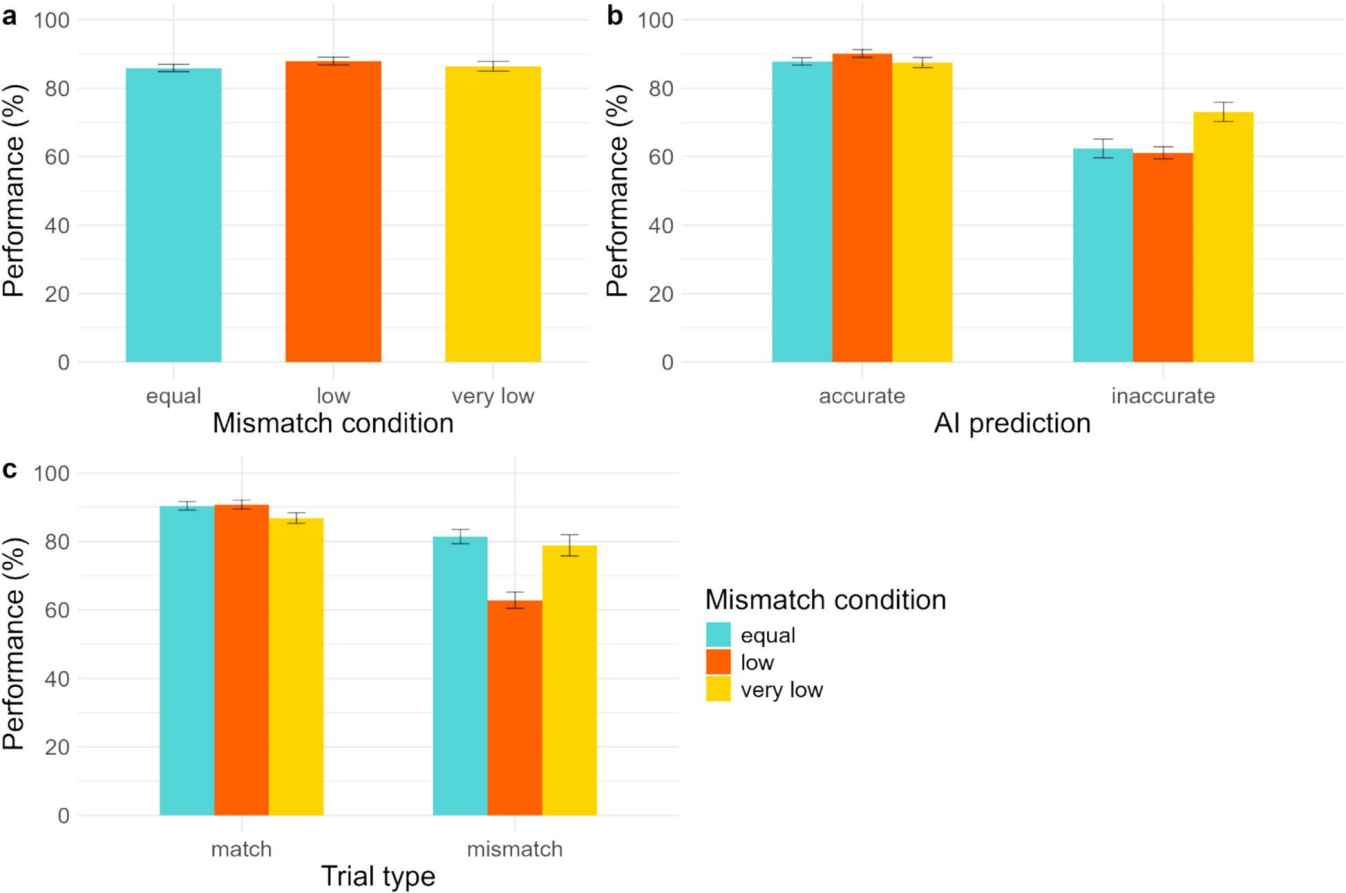
Overall mean performance in (**a**) the three mismatch conditions, (**b**) each mismatch condition by accuracy of AI prediction, and (**c**) each mismatch condition by trial type. Error bars represent standard error

Participants’ mean performance in each of the three mismatch frequency conditions is provided in Fig. 4a. A logistic regression using a mixed-effects model was conducted for the dependent variable performance, which was regressed on mismatch frequency, AI prediction accuracy, and the interaction of the two as fixed factors and participants and face pairs added as random factors (Table 4). As in Experiment 1, there was a main effect of AI prediction accuracy, with participants performing worse when receiving an *inaccurate* prediction (see Fig. 4b), though they overall tended to follow *inaccurate* predictions less (Table S6 in supplementary material). We did not find a significant effect of mismatch frequency on performance or an interaction effect between mismatch frequency and AI prediction accuracy. We found that 13, 26, and 29 people in the *equal*, *low*, and *very low mismatch* conditions, respectively, exceeded the performance of the AI. We report on the results for AI usefulness, confidence, and task difficulty in the supplementary material. A limitation of the present experiment is that the face pairs were not counterbalanced across the conditions due to the incongruency of numbers. Further, the limited number of mismatch trials in the low and very low conditions means that average performance would be greatly influenced by the difficulty of the limited number of face pairs.

**Table 4:** Results of the mixed-effects logistic regression (Experiment 2)

	Performance (Model 1)					Performance (Model 2)				
Predictors	Odds ratios	SE	95% CI	Statistic	*p*	Odds ratios	SE	95% CI	Statistic	*p*
Intercept	11.98	2.52	7.93–18.11	11.78	** < 0.001**	16.70	4.61	9.72–28.67	10.21	** < 0.001**
Mismatch condition [low]	1.30	0.34	0.77–2.18	0.98	0.326	1.00	0.32	0.53–1.87	− 0.01	0.990
Mismatch condition [very low]	1.08	0.28	0.64–1.81	0.28	0.781	0.71	0.22	0.38–1.32	− 1.09	0.278
AI prediction [inaccurate]	0.20	0.14	0.06–0.76	− 2.38	**0.017**					
Mismatch condition [low] x AI prediction [inaccurate]	0.57	0.54	0.09–3.61	− 0.60	0.547					
Mismatch condition [very low] x AI prediction [inaccurate]	1.26	1.18	0.20–7.89	0.25	0.806					
trial type [mismatch]						0.41	0.15	0.20–0.83	− 2.47	**0.014**
Mismatch condition [low] x trial type [mismatch]						0.26	0.18	0.07–1.02	− 1.94	0.053
Mismatch condition [very low] x trial type [mismatch]						1.43	1.27	0.25–8.17	0.40	0.690

Model 1: Mismatch condition x AI prediction accuracy; Model 2: Mismatch condition x Trial type

**Model 1:** Random effects:* σ*^2^ = 3.29, τ_00 TrialID_ = 1.11, τ_00 participant_ = 0.71, ICC = 0.36,* N*_TrialID_ = 120, *N*_participant_ = 78, observations = 9360, marginal *R*^2^ = 0.041/conditional *R*^2^ = 0.382. **Model 2:** Random effects: *σ*^2^ = 3.29, τ_00 TrialID_ = 1.11, τ_00 participant_ = 0.71, ICC = 0.36, *N*_TrialID_ = 120, *N*_participant_ = 78, observations = 9360, marginal *R*^2^ = 0.042/conditional *R*^2^ = 0.383. OR > 1 is associated with higher odds for correct decision; OR < 1 is associated with lower odds for correct decision. The respective intercepts indicate that the probability of a correct decision was 0.92, & 0.94 in models 1 & 2, respectively. Including ‘mismatch frequency’ and ‘AI prediction accuracy’ (Model 1) improved model fit over the null model and the model with ‘mismatch frequency’ only (χ^2^(3) = 18.57, *p* < 0.001). Similarly, including ‘mismatch frequency’ and ‘trial type’ (Model 2) improved model fit over the null model and ‘mismatch frequency’ only model (χ^2^(3) = 19.17, *p* < 0.001)

A second mixed-effects logistic regression was conducted with mismatch frequency and trial type (*match* or *mismatch*) as predictors (Table 4). Trial type was specifically included as a predictor in this experiment, as we were interested in whether detection of *matches* or *mismatches* was disproportionately affected as a result of unequal distribution of the trial types. There was a main effect of trial type, with participants performing worse on *mismatch* trials compared to *match* trials. The interaction between mismatch frequency and trial type was not found to be statistically significant.

These findings are in line with the results from two previous studies (Baker & Bindemann, 2025; Bindemann et al., 2010), which also found no effect of mismatch frequency on accuracy, albeit in a scenario without the involvement of AI. The current experiment extends those findings to scenarios involving AI in the decision-making process. The findings indicate that users adjust their expectations and decision-making behaviour according to the mismatch frequency. Papesh and Goldinger (2014), on the other hand, found that performance was negatively influenced by lower mismatch frequency. In the procedure used in Papesh and Goldinger, participants were provided feedback on the accuracy of their decisions, which was not the procedure in our study or the other two studies that also did not find an effect on performance (Baker & Bindemann, 2025; Bindemann et al., 2010).

Another study that included AI predictions reported a response bias towards match (Mueller et al., 2024) and lower accuracy on mismatch trials. However, the study did not directly compare low and balanced mismatch frequency designs, and they informed participants of the mismatch rate, which we did not. We found that participants were consistently worse on mismatch trials across all mismatch frequencies, so not necessarily as a result of the base rate. Further, computation of sensitivity (d’) and criterion (C) revealed little to no bias in the *equal* condition, but similar to Mueller et al., a considerable liberal bias was observed in both conditions with *low* mismatches (see Supplementary material). These findings are in line with previous studies (Baker & Bindemann, 2025; Papesh & Goldinger, 2014; Stabile et al., 2024), which also reported a shift in criterion in low prevalence conditions.

## Experiment 3

The previous experiments employed only a binary AI prediction-*match* or *mismatch*, and participants only made a binary matching decision. In this experiment, we wanted to examine whether providing additional explanatory information about the binary AI prediction influenced performance. The binary match/mismatch prediction provided by the algorithm is produced by comparing the two faces, generating a similarity score for the faces, and then using a pre-defined threshold to classify the pair as a match or mismatch. Previously, two studies (Carragher & Hancock, 2023; Mueller et al., 2024) examined how providing this similarity score influences performance. Carragher and Hancock provided the similarity scores in addition to the binary decision, whereas Mueller et al. presented only the similarity scores. Both studies did not find an overall performance advantage of providing similarity scores. However, the study by Mueller et al. employed a 6-point rating scale for recording the response, as opposed to the binary response type in Carragher and Hancock, and found that responses were less biased when participants saw similarity scores instead of binary predictions.

In this experiment, we combine two aspects from these two studies, i.e. providing similarity scores simultaneously with binary predictions and employing a 6-point response scale to assess the impact of the explanatory information on participants’ certainty in their decision and on their performance. Additionally, we also aim to examine how it interacts with AI prediction accuracy and assess whether it helps mitigate overreliance on inaccurate predictions.

### Participants

Participants were recruited from Prolific and received monetary compensation in exchange for their participation. Eighty people took part in the study. Outliers (based on boxplots), people performing below chance (< 50% accuracy), failing attention checks, or finishing unrealistically quickly (average response time < 1 s), were excluded, resulting in 75 participants included in the final analysis. The demographic characteristics of our sample from Experiment 3 are presented in Table 5.

**Table 5:** Demographic characteristics of participants in Experiment 3

	Overall
	(*N* = 75)
Age	
Mean (SD)	32.9 (10.2)
Median [Min, Max]	30.0 [18.0, 63.0]
Gender	
Female	23 (30.7%)
Male	49 (65.3%)
Non-binary	2 (2.7%)
Transgender	1 (1.3%)
Country	
Germany	75 (100%)
Ethnicity	
East Asian	1 (1.3%)
Middle Eastern/Arab	3 (4.0%)
White/Caucasian	70 (93.3%)
Other/Mixed	1 (1.3%)

### Stimuli and procedure

**Fig. 5:**
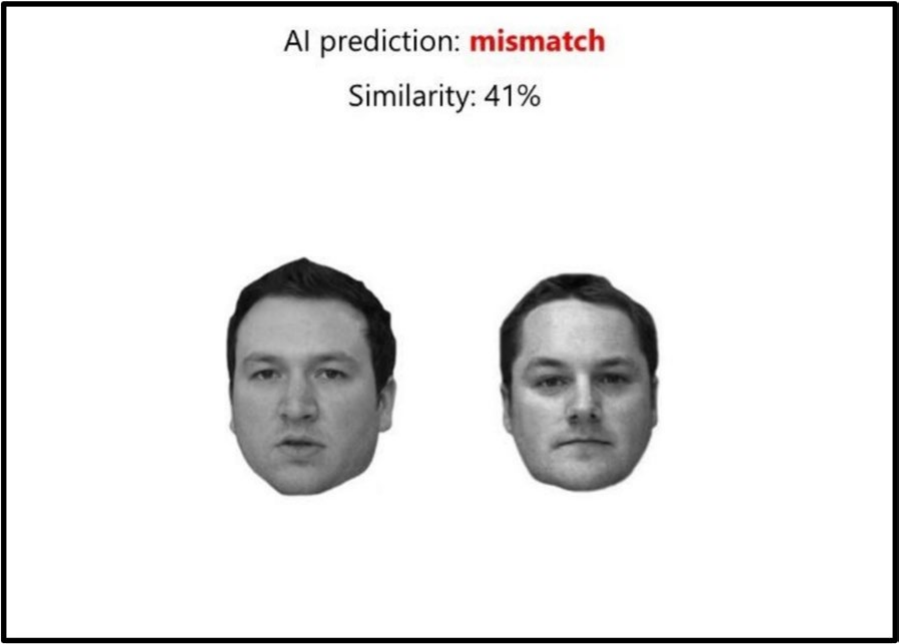
Example of stimuli with similarity rating in addition to the binary AI prediction (Experiment 3)

As in the previous experiments, a binary match or mismatch prediction was presented. Additionally, in half of the trials (i.e. in two blocks), a ‘similarity rating’, expressed as the percentage of similarity between the two images was presented (see Fig. 5 for an example). It was obtained by inverting the ‘distance’ value, ranging from 0 to 1, which the algorithm calculated to make the matching prediction (see General Method) and showing it as a percentage, for ease of understanding. For example, if the ‘distance’ calculated by the algorithm was 0.75, the similarity value would be 25%. The stimuli were presented in four blocks, with each block consisting of 40 trials. Two versions of the experiment were created to counterbalance the order of presentation of the two experimental conditions. Within each block, the order of presentation of face pairs was completely randomised. In order to assess certainty in the decision, the binary decision format from previous experiments was replaced by a 6-point scale representing certainty in the match or mismatch decision (1 = *definite mismatch*, 2 = *likely mismatch*, 3 = *guess mismatch*, 4 = *guess match*, 5 = *likely match*, 6 = *definite match*), which was presented on the screen. The response was registered by a mouse click on the scale. Participants did not receive any information regarding the accuracy rate of the AI. Six trials in each condition (3 per block) were paired with inaccurate advice. Three inaccurate trials in each condition were identity matches and three were identity mismatches and were distributed over the two blocks in a 2:1, 1:2 ratio.

### Results and discussion

A 2 (advice condition: binary only vs binary + similarity prediction) × 2 (AI prediction accuracy: accurate vs inaccurate prediction) within-subject design was employed.

#### Performance

**Fig. 6:**
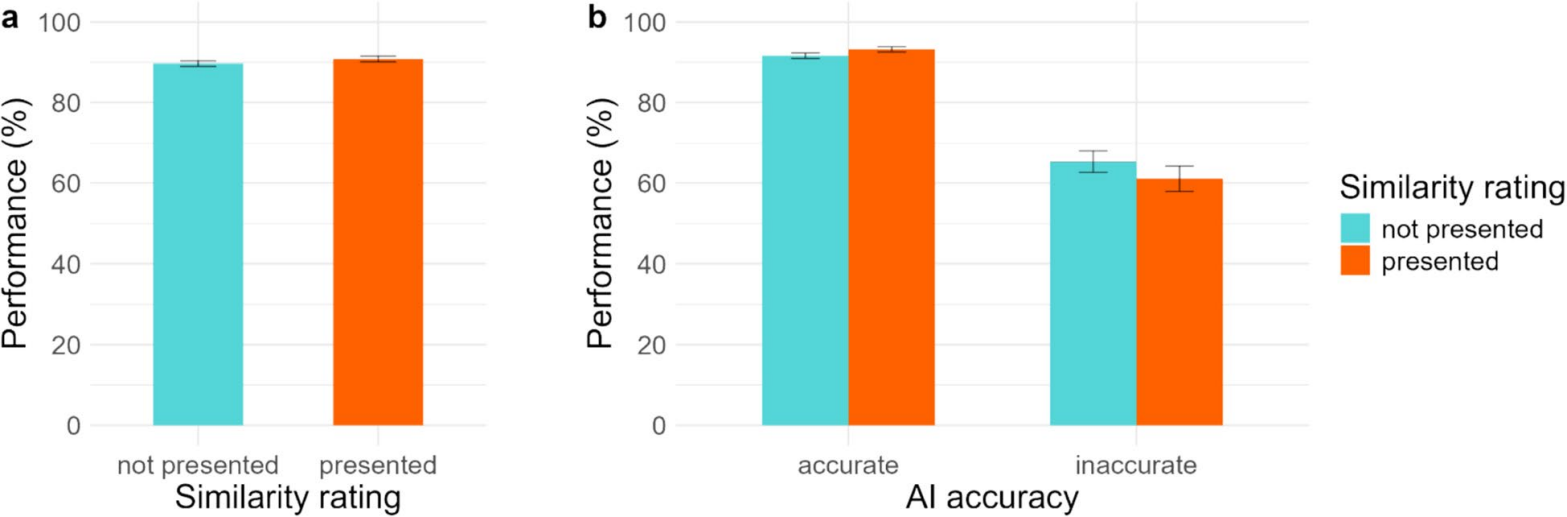
Overall performance in (**a**) *binary only* and *binary* + *similarity rating* advice conditions and (**b**) interaction between advice conditions and AI prediction accuracy. Error bars represent standard error

The response ratings were converted to a binary match or mismatch response by splitting the scale in half. These responses were then coded as correct or incorrect. An overview of mean performance is presented in Fig. 6. A mixed-effects logistic regression for performance was conducted, with advice condition, AI prediction accuracy and their interaction as fixed factors, and participants and face pairs as random factors. Participants performed better when presented similarity ratings than when they only saw binary advice (Table 6). The mean performance, however, was only 1.13% higher with the similarity ratings. Similar to previous experiments, AI prediction accuracy affected performance significantly, with performance being 29.2% lower when inaccurate predictions were presented. The interaction between the advice conditions and the accuracy of the prediction did influence performance. Pairwise comparisons showed that when an *inaccurate* prediction was presented, performance in the two advice conditions did not differ significantly (*p* = 0.102). However, when an *accurate* prediction was presented, participants performed better when presented the similarity ratings compared to only binary advice (*p* < 0.001). The findings from the SDT analysis (see supplementary material) suggest that this may be due to increased discriminability, as sensitivity was higher when similarity ratings were presented for accurate predictions. It was assessed how many participants exceeded the accuracy of AI and we found that 26 participants in the binary only prediction and 33 participants in the binary + similarity rating prediction condition exceeded the AI’s accuracy.

**Table 6:** Results of the mixed-effects logistic regression (Experiment 3)

	Performance				
Predictors	Odds Ratios	SE	95% CI	Statistic	*p*
Intercept	26.32	4.25	19.19–36.12	20.27	** < 0.001**
Similarity ratings [presented]	1.36	0.11	1.16–1.58	3.92	** < 0.001**
AI prediction [inaccurate]	0.09	0.04	0.03–0.22	− 5.12	** < 0.001**
Similarity ratings [presented] × AI prediction [inaccurate]	0.54	0.11	0.37–0.81	− 3.03	**0.002**

Random effects: *σ*^2^ = 3.29, τ_00 TrialID_ = 2.22, τ_00 participant_ = 0.42, ICC = 0.45, *N*_TrialID_ = 160, *N*_participant_ = 75, observations = 12,000, marginal *R*^2^ = 0.084/conditional *R*^2^ = 0.492. OR > 1 is associated with higher odds for correct decision; OR < 1 is associated with lower odds for correct decision. The intercept indicates that the probability of a correct decision was 0.96 when all predictors are zero. The model fit significantly improved over the null model and the ‘similarity ratings’ only model when ‘similarity ratings’ and ‘AI prediction accuracy’ were included (χ^2^(3) = 48.40, *p* < 0.001)

Overall, presenting similarity ratings appears to show some benefit, albeit limited, in terms of improving the performance of humans, as it increased overall reliance on AI predictions (see Table S10 in supplementary material). Previous studies that examined the effect of presenting similarity values did not find a significant advantage over binary predictions alone on performance (Carragher & Hancock, 2023; Mueller et al., 2024). Interestingly, we did not observe any benefit of presenting similarity ratings for inaccurate advice cases in our study. Instead of mitigating errors committed by AI, the mean performance on trials with inaccurate AI prediction was slightly lower with similarity ratings than binary advice, though not significantly different.

#### Response certainty

Next, to evaluate the effect of binary only vs. binary + similarity rating AI predictions on participants’ level of certainty in their decision, participants’ responses on the 6-point scale were collapsed into three categories of certainty: (1) guess, likely, definite-, by dropping the match or mismatch aspect of the response. A mixed-effects ordinal logistic regression for certainty, with advice condition and AI prediction accuracy as predictors, and participants and face pairs as random factors, showed there was an effect of advice condition on certainty (Table 7). Participants were more likely to respond with a *likely* (*p* = 0.015), and less likely to respond with *definitely* (*p* = 0.011) when only the binary prediction was presented. This pattern is somewhat different to what was observed in Mueller et al. (2024), where participants’ responses on a similar 6-point scale were moderated by the similarity value, when displayed, whereas the bias in the direction of the AI prediction was similar for all pairs in the binary prediction condition. However, while the binary prediction condition was the same in both studies, the important difference here was that in our study we presented the similarity scores in addition to the binary prediction, whereas Mueller et al. presented only the similarity scores, pointing towards an important consideration while designing the interaction with AI aids. Similarity scores, when paired with a binary prediction, appear to increase confidence in the binary prediction itself. When presented alone, however, they prompt users to engage in more evaluative judgment.

**Table 7:** Results of the mixed-effects ordinal regression for decision certainty (Experiment 3)

	Certainty				
Predictors	Odds Ratios	SE	95% CI	Statistic	*p*
Similarity ratings [presented]	1.17	0.05	1.08–1.27	3.94	** < 0.001**
AI prediction [inaccurate]	0.42	0.12	0.24–0.74	− 2.99	**0.003**
Similarity ratings [presented] × AI prediction [inaccurate]	1.02	0.14	0.77–1.34	0.13	0.896

Random effects: *σ*^2^ = 3.29, τ_00 TrialID_ = 0.81, τ_00 participant_ = 1.30, ICC = 0.39, *N*_TrialID_ = 160, *N*_participant_ = 75, observations = 12,000, marginal *R*^2^ = 0.01/conditional *R*^2^ = 0.398. OR > 1 is associated with higher odds for increased certainty; OR < 1 is associated with lower odds for increased certainty. The model fit significantly improved over the null model and the ‘similarity ratings’ only model when ‘similarity ratings’ and ‘AI prediction accuracy’ were included (LR(3) = 26.27, *p* < 0.001)

Further, an effect of AI prediction accuracy was also observed. Participants were less likely to respond *guess* (*p* = 0.019) or *likely* (*p* < 0.001) and more likely to respond *definite* (*p* < 0.001) when an accurate prediction was presented. The interaction between the two predictors was not significant. Overall, providing similarity ratings did increase participants’ certainty in their decisions. It is interesting to note that while participants’ response patterns indicate more certainty in their decisions in the condition with similarity ratings, this is not reflected in their self-reported confidence or task difficulty rating, nor do they rate the AI prediction with similarity ratings to be more useful. We report on the confidence, AI usefulness, and task difficulty findings in detail in the supplementary material.

**Fig. 7:**
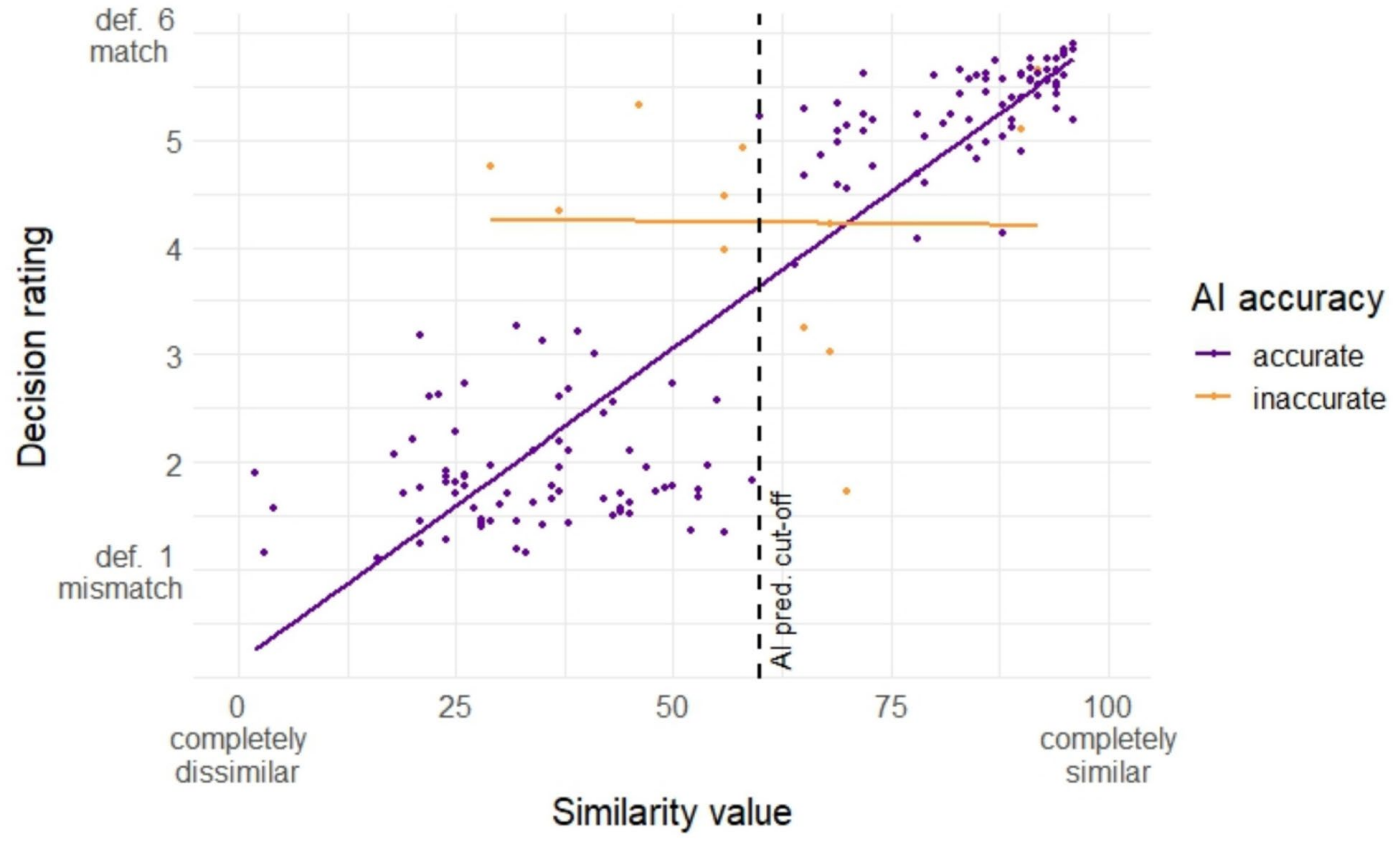
Relationship between similarity value and match/mismatch certainty rating by accuracy of AI prediction

Additionally, we evaluated the association between the AI predicted degree of similarity between the face pairs and participants’ certainty in their decision (Fig. 7). We only included trials where similarity value was presented and conducted a mixed-effects ordinal logistic regression (Table 8), with similarity and AI prediction accuracy as fixed factors, and participants as a random factor. Both main effects and their interaction were significant. For accurate AI predictions, an increase in the degree of similarity was significantly associated with an increase in the odds of participants’ response in the direction of a definite match, in line with the pattern observed in Mueller et al. (2024). This was not observed for inaccurate AI predictions. It is also noteworthy here that the degree of similarity for the inaccurate AI predictions tended to cluster around the cut-off margin.

**Table 8:** Results of the mixed-effects ordinal regression for response rating as a result of similarity value and AI prediction accuracy (Experiment 3)

	Response rating				
Predictors	Odds ratios	SE	95% CI	Statistic	*p*
Similarity	1.08	0.00	1.08–1.09	58.29	** < 0.001**
AI prediction [inaccurate]	229.17	72.83	122.92–427.25	17.10	** < 0.001**
Similarity × AI advice [inaccurate]	0.92	0.00	0.92–0.93	− 15.63	** < 0.001**

Random effects: *σ*^2^ = 3.29, τ_00 participant_ = 0.22, ICC = 0.06, *N*_participant_ = 75, observations = 6000, marginal *R*^2^ = 0.572/conditional *R*^2^ = 0.599. The model fit was significantly better than the null model and the ‘similarity ratings’ only model when ‘similarity ratings’ and ‘AI prediction accuracy’ were included (LR(2) = 519.11, *p* < 0.001)

Overall, our findings suggest that similarity rating offers some advantage, as it increased certainty in the decision compared to the binary predictions. The degree of similarity reported by the AI was also directly associated with certainty. However, performance-wise, the impact of similarity ratings appears to be modest, as participants’ performance improved only marginally, and importantly, did not improve when the prediction was inaccurate.

## General discussion

The increasing use of AI-enabled decision aids necessitates the assessment of factors influencing their effective integration within existing systems (Gaube & Lermer, 2023; Hudecek et al., 2024). To this end, we conducted a series of experiments to investigate design factors influencing participants’ face matching performance while using an AI-enabled decision aid.

First, we found that, when stated AI accuracy was high or unknown, participants performed significantly better than when no predictions were presented. In the three conditions with AI predictions, although there were no significant differences in performances between these conditions, the highest mean performance observed was for the unknown accuracy condition. We did not find a significant interaction between these conditions and prediction accuracy either. Although not statistically significant, observation of participants’ response patterns indicated a tendency to accept inaccurate predictions when high AI accuracy was stated, and a tendency to dismiss accurate predictions when low AI accuracy was stated. These results are largely in line with the findings from Carragher and Hancock (2023), in which participants were presented three conditions: no predictions, high accuracy AI, and low accuracy AI, and only half of the participants were informed of the AI’s accuracy. The AI accuracy reported to the participants was the same as the actual accuracy, unlike in our study. The study found a significant improvement in performance in the high accuracy rate condition in comparison with the no predictions condition, and comparable performance on the low accuracy rate condition and the control (no prediction) condition. Furthermore, participants that were not informed of the respective high and low accuracy rates, still performed comparably to the respective groups that were informed of the accuracy rates, indicating that users can and do operate intuitively, and may be able to approach the decision in a more non-biased manner when not informed of the AI’s accuracy. It is common practice to promote an AI-enabled decision aid based on the algorithm’s accuracy (Grother et al., 2021). However, taken together with the findings from Carragher and Hancock, our findings suggest that revealing the AI’s accuracy does not necessarily provide any advantage.

Second, we found that the ratio of match and mismatch pairs did not influence performance or reliance on AI predictions but did result in a response bias towards *match*. This ratio usually tends to be skewed towards fewer mismatches in real-world settings. This is not only the case with face matching, where for example, border control officers would encounter a mismatch identity far less frequently than the 50–50 ratio seen in experiments, but also extends to other domains such as medicine. For example, in chest X-rays, cases with no abnormalities tend to outnumber cases with abnormalities quite significantly (Wang et al., 2017). Although this is not a variable that can be altered in reality, it is important to understand whether it influences the performance of humans.

Third, presenting similarity ratings in addition to a binary match/mismatch prediction provided limited benefit in enhancing performance, i.e. performance (and discriminability) improved only when advice was correct. Some previous studies in other domains that have provided additional information in the form of explainable predictions similarly found very limited benefit (Cecil et al., 2023; Grimmelikhuijsen, 2023; Selten et al., 2023), while studies in the face matching domain that similarly provided similarity ratings found no benefit in terms of performance (Carragher & Hancock, 2023; Mueller et al., 2024). Importantly, performance on inaccurate AI prediction cases did not improve as a result of providing similarity ratings. Further, the AI-generated similarity value predicted participants’ response pattern for accurate AI prediction cases, but not for inaccurate prediction cases. The similarity values for inaccurate predictions also mostly clustered around the cut-off score. We also found that presenting similarity ratings increased users’ certainty in their decision. Some previous studies have reported higher levels of trust when additional information or an explanation for an algorithm's prediction was presented (Bansal et al., 2021; Bussone et al., 2015), even when the explanations are misleading (Lakkaraju & Bastani, 2020; Sadeghi et al., 2024). In our study, although we did not find performance to be significantly better when inaccurate prediction with similarity ratings were presented, we also did not find performance to be significantly worse. The similarity ratings generated by the algorithm which were presented to participants did not actually explain the binary AI prediction, and it may be that it simply did not provide enough added value beyond the binary prediction. Rather, the presence of this additional information simply increased trust in the AI’s judgement or capability, thereby increasing reliance on AI predictions, irrespective of whether they were correct or incorrect.

Finally, across all experiments, we consistently observed a steep decline in performance when inaccurate AI predictions were presented, indicating a substantial overreliance on the AI aid. This was accompanied by a lower sensitivity on trials with inaccurate predictions. Interestingly, inaccurate predictions also generally elicited a higher liberal response bias, except for Experiment 2, where a liberal bias was observed for the accurate predictions cases in both conditions with low mismatch frequency. The strong effect of inaccurate AI advice on performance raises the question of whether presenting a-priori predictions to users is appropriate or if it might bias their judgement too much. Preliminary evidence indicates that making a decision independently and only receiving the AI prediction as a second opinion reduces the risk of overreliance on inaccurate AI advice (Buçinca et al., 2021; Noti & Chen, 2022). Further studies in different fields should be conducted to confirm this evidence. This might be especially useful for high-risk decision-making scenarios. Expanding on this idea, it is crucial to consider the context in which decisions are made. In the present experiment, incorrect decisions bore no adverse consequences, which would not be the case in most real-world applications. Participants in experimental settings may show a greater tendency to agree with the AI due to the low incentive to exercise heightened scrutiny. Preliminary evidence shows that overreliance is reduced in situations involving incentives (Vasconcelos et al., 2023). Again, more research is needed to understand the context in which overreliance on inaccurate AI predictions appears and how it can be mitigated.

It is noteworthy, that despite displaying a strong tendency to align with the AI prediction, participants’ performance was not perfect when accurate predictions were presented. This means that participants also rejected accurate predictions. Consequently, on a group level, the combined performance of humans and the AI did not exceed that of the AI alone. Nevertheless, there were instances where the human–AI team accomplished this, and certain participants achieved perfect performance. It is well established that individual face identification ability varies widely (Bindemann et al., 2012; Kokje et al., 2018). In the current study, there was a ceiling effect for the top performers, possibly due to the stimuli being easy, so we could not test whether individuals in the higher end of the spectrum of face matching ability are able to achieve perfect or near-perfect performance when teamed with an AI. However, Carragher et al. (2024) reported in their study that participants who achieved the same or better aided performance than the AI already had better baseline (unaided) performance compared to individuals who did not. It would also be of interest to investigate whether those who perform jobs involving face identification and interact with AI systems regularly for it, are able to achieve a better performance than those who do not.

### Practical implications

Currently, it appears that humans are the weaker link, with algorithms outperforming the majority of the humans even when teamed with the algorithm. However, while AI algorithms have achieved remarkable levels of accuracy in various domains, even high-performing algorithms make fundamental errors, such as declaring a face pair a match when the pairs may belong to two different races or may be of different sexes (Hancock et al., 2020). Additionally, algorithms are only as good as the data they have been trained on, and racial and gender bias is prevalent in facial recognition algorithms (Khalil et al., 2020). Therefore, human involvement is still often warranted, particularly in ambiguous cases. For example, the algorithm used in the present study had a cut-off similarity value for determining matches and mismatches. The face pairs with distance values close to the cut-off were not treated ambiguously by the system despite more of the inaccurate cases being clustered around the cut-off score. Deferring only the cases to humans in which the system lacks certainty might be a good strategy to improve human–AI-teams (Bondi et al., 2022). Another strategy, as discussed above, may be to provide AI predictions only after a first decision is made by humans or only upon request, which is being explored in emerging face matching literature (Hua et al., 2024; Kokje et al., 2025; Mueller et al., 2024). Preliminary evidence suggests that while overall accuracy is not significantly affected by this method, it does influence patterns of reliance on AI and confidence in the decision and is a strategy worth exploring further.

The current study shows us that how well humans utilise AI predictions can change by changing aspects of the design and presentation, and performance can be improved. Although we employed the one-to-one face matching paradigm in the current study, some of the user-related challenges, when using AI-enabled decision aids, are likely to transcend fields and applications. Future studies should endeavour to examine design factors further in the directions discussed here.

### Limitations and future directions

A limitation of the study is the participant group. The study was conducted with random samples, and not with groups of people with a specific level of expertise. The group also may or may not have consisted of individuals who are end-users of face matching AI systems or do identity matching on a regular basis professionally. However, previous evidence indicates that people who conduct face matching professionally (e.g. passport officers), including those with extensive experience, are not necessarily better at the task than the general population (White et al., 2014). This, however, does not account for long-term experience using an AI tool for face matching, which may influence how well users are able to utilise AI predictions and understand the limitations of the technology. Additionally, it also does not account for super-recognisers or people with specialised training, such as forensic face examiners, who have been shown to outperform untrained individuals (Phillips et al., 2018). The experimental design also introduces a limitation stemming from the low-risk nature of the decision-making task, attributed to the absence of negative consequences for poor performance as well as a positive incentive for good performance. Consequences may introduce behavioural differences, as has been observed in some face matching studies (without AI), where participants made fewer errors, when errors resulted in negative consequences (Stabile et al., 2024; Yuen & Fitzgerald, 2024).

In Experiment 2, we were unable to counterbalance the stimuli with the experimental conditions, therefore, it cannot be ruled out that the effects observed resulted from difficulty of stimuli itself. Further, the stimuli used in all the experiments did not include female faces. Additional studies which include female faces are required to increase the generalizability of these findings. Another limitation is the limited number of trials with inaccurate AI predictions. We used actual predictions from an algorithm in our study, and the accuracy rate of the algorithm, to have the study design closer to real-world conditions. While this approach enhances the ecological validity of the study, it resulted in a limited number of inaccurate AI prediction trials compared to accurate prediction trials, which may have limited the statistical power to detect effects in this condition.

Finally, the stimuli and experimental set-up mimics the best-case scenario for identity verification compared to real-world settings, which reduces the external validity of the study. The stimuli depicted two pictures taken minutes apart, whereas, in real life, photo IDs are often used for a decade or sometimes longer, which means the appearance of individuals can differ rather drastically, making identification more difficult. Additionally, participants in the study performed the face matching task for a short period of time with breaks in between. In the real world, users could be performing the task for hours on end, and factors like fatigue and attention may negatively impact their performance (Alenezi et al., 2015; Fysh & Bindemann, 2017; Megreya et al., 2013), which may also result in increased overreliance on AI aids.

## Conclusion

In conclusion, the findings of this study put forward important considerations concerning the design of human–AI collaborative systems which may influence humans’ use of AI advice, and consequently, their performance. Our findings suggest no advantage of revealing the accuracy of AI systems to users. Additionally, our findings showed that having a lower base rate of mismatches elicits a response bias. Further, we found that presenting information regarding the AI’s perceived similarity of the face pairs in addition to the binary AI predictions provided limited benefit, and note that it increased overall reliance on AI, and importantly, did not really help to decrease overreliance on incorrect advice. Thus, it did not provide added value in detecting incorrect advice. Finally, in the current study, the human–AI team overall did not outperform the AI alone. Further follow-up studies are required to refine aspects of the design that may optimise performance of the human–AI team.

## Supplementary Information

Additional file 1.

## Data Availability

The datasets supporting the conclusions of this article are available in an OSF repository. All the experiments were pre-registered.

## Abbreviations

AI: Artificial intelligence
EMM: Estimated marginal means
GUFD: Glasgow University Face Database
SDT: Signal detection theory
DCNN: Deep convolutional neural network

## Footnotes

1 In order to avoid confusion, from here on, we use the term ‘performance’ to refer to accuracy of participants and ‘accuracy’ to refer to accuracy of AI.
