# Supplementary material for: Understanding the influence of design-related factors on human–AI teaming in a face matching task
Source: Cogn Res Princ Implic. 2026 Jan 7;11:4. doi: 10.1186/s41235-025-00701-x (PMC12779867; doi:10.1186/s41235-025-00701-x)
Supplement: Supplementary file 1 — Additional file 1. [file 41235_2025_701_MOESM1_ESM.docx]

# Supplementary material

# Analyses

#### Signal Detection Theory (SDT)

We also examined the data in the context of SDT, which separates individuals’ sensitivity to facial identity from their bias in decision-making. We calculated the hit (match trials correctly classified as matches) and false alarm (mismatch trials incorrectly classified as matches) rates. Based on that, in accordance with Stanislaw and Todorov (1999), we computed the sensitivity (d’), which measures how well participants can differentiate signal from noise, and criterion (C), which is a measure of response bias. Extreme values of 0 or 1 in hit and false alarm rates were adjusted, wherein 0 and 1 were replaced with 0.5/*n* and (n-0.5)/*n,* respectively, where *n* is the number of signal or noise trials, respectively. We conducted ANOVAs to compare the d’ and C across conditions, and followed up significant findings with pairwise comparisons using a Bonferroni adjustment.

# Results

| **Experiment 1** |  |  | | |  | |  | | |  | |  |  |
| --- | --- | --- | --- | --- | --- | --- | --- | --- | --- | --- | --- | --- | --- |
| *Stated AI accuracy* | *Control* | | | *High (95%)* | | | | *Low (70%)* | | | *Unknown* | | |
| Mean | 87.63 | | | 89.56 | | | | 88.43 | | | 90.03 | | |
| SD | 7.74 | | | 6.08 | | | | 8.01 | | | 6.11 | | |
|  |  | | |  | | | |  | | |  | | |
| *Stated AI accuracy* | *High (90%)* | | | | | *Low (70%)* | | | | *Unknown* | | | |
| *AI prediction* | *accurate* | | *inaccurate* | | | *accurate* | | | *inaccurate* | *accurate* | | *inaccurate* | |
| Mean | 91.21 | | 66.67 | | 89.85 | | | | 68.09 | 91.18 | | 73.23 | |
| SD | 5.88 | | 30.06 | | 8.03 | | | | 27.1 | 5.91 | | 25.48 | |
|  |  |  | | |  | |  | | |  | |  |  |
|  |  |  | | |  | |  | | |  | |  |  |
| **Experiment 2** |  |  | | |  | |  | | |  | |  |  |
| *Mismatch condition* | *Equal* | | | | *Low* | | | | | *Very low* | | |  |
| Mean | 85.9 | | | | 87.95 | | | | | 86.41 | | |  |
| SD | 9.28 | | | | 9.81 | | | | | 12.7 | | |  |
|  |  |  | | |  | |  | | |  | |  |  |
| *Mismatch condition* | *Equal* | | | | *Low* | | | | | *Very low* | | |  |
| *AI prediction* | *accurate* | *inaccurate* | | | *accurate* | | *inaccurate* | | | *accurate* | | *inaccurate* | |
| Mean | 87.8 | 62.39 | | | 90.12 | | 61.11 | | | 87.49 | | 73.08 |  |
| SD | 9.65 | 24.24 | | | 10.2 | | 15.59 | | | 12.91 | | 24.65 |  |
|  |  |  | | |  | |  | | |  | |  |  |
| *Mismatch condition* | *Equal* | | | | *Low* | | | | | *Very low* | | |  |
| *Trial type* | *match* | *mismatch* | | | *match* | | *mismatch* | | | *match* | | *mismatch* |  |
| Mean | 90.38 | 81.41 | | | 90.74 | | 62.82 | | | 86.81 | | 78.85 |  |
| SD | 11.01 | 18.43 | | | 11.27 | | 20.84 | | | 13.67 | | 27.35 |  |
|  |  |  | | |  | |  | | |  | |  |  |
|  |  |  | | |  | |  | | |  | |  |  |
| **Experiment 3** |  |  | | |  | |  | | |  | |  |  |
| *Similarity ratings* | *Not presented* | | | | *Presented* | | | | |  | |  |  |
| Mean | 89.67 | | | | 90.8 | | | | |  | |  |  |
| SD | 5.86 | | | | 6.21 | | | | |  | |  |  |
|  |  |  | | |  | |  | | |  | |  |  |
| *Similarity ratings* | *Not presented* | | | | *Presented* | | | | |  | |  |  |
| *AI prediction* | *accurate* | *inaccurate* | | | *accurate* | | *inaccurate* | | | | |  |  |
| Mean | 91.64 | 65.33 | | | 93.21 | | 61.11 | | |  | |  |  |
| SD | 5.83 | 23.21 | | | 5.7 | | 27.31 | | |  | |  |  |

**Table S1**: Means and SDs in different conditions in Experiments 1, 2, and 3.

# Experiment 1

## Results

### AI usefulness, confidence, and difficulty

We were interested in whether there were any differences in participants’ self-reported ratings for usefulness of the AI predictions, confidence in their decisions, and difficulty of the task (Figure S1), as a result of the four conditions introduced. Three mixed-effects linear regressions were conducted, one for each of the three variables, with stated accuracy as fixed factor and participants as random factor. We found a significant effect of stated accuracy on AI usefulness (see Table S2). Post hoc pairwise comparisons showed that ratings were higher in the high stated accuracy condition compared to the low (*p* = .001) and unknown (*p* < .001) conditions. For confidence and difficulty ratings, we did not find statistically significant effects (see Table S3).

|  | **AI usefulness** | | | | |
| --- | --- | --- | --- | --- | --- |
| *Predictors* | *Estimates* | *SE* | *95% CI* | *Statistic* | *p* |
| Intercept | 6.59 | 0.21 | 6.18 – 6.99 | 32.07 | **<0.001** |
| Condition [70%] | -0.83 | 0.23 | -1.29 – -0.36 | -3.52 | **0.001** |
| Condition [Unknown] | -1.03 | 0.23 | -1.49 – -0.57 | -4.40 | **<0.001** |
| **Random Effects** | | | | | |
| σ^2^ | 2.54 | | | | |
| τ_00_ _participant_ | 1.34 | | | | |
| ICC | 0.35 | | | | |
| N _participant_ | 92 | | | | |
| Observations | 276 | | | | |
| Marginal R^2^ / Conditional R^2^ | 0.049 / 0.378 | | | | |

**Table S2**: Results of the mixed-effects linear regression for AI usefulness ratings (Experiment 1).

*Notes: The fit of the model was significantly better than the null model when ‘stated AI accuracy condition’ was included (χ²*(2) = 20.68, *p* < .001)*.*

**
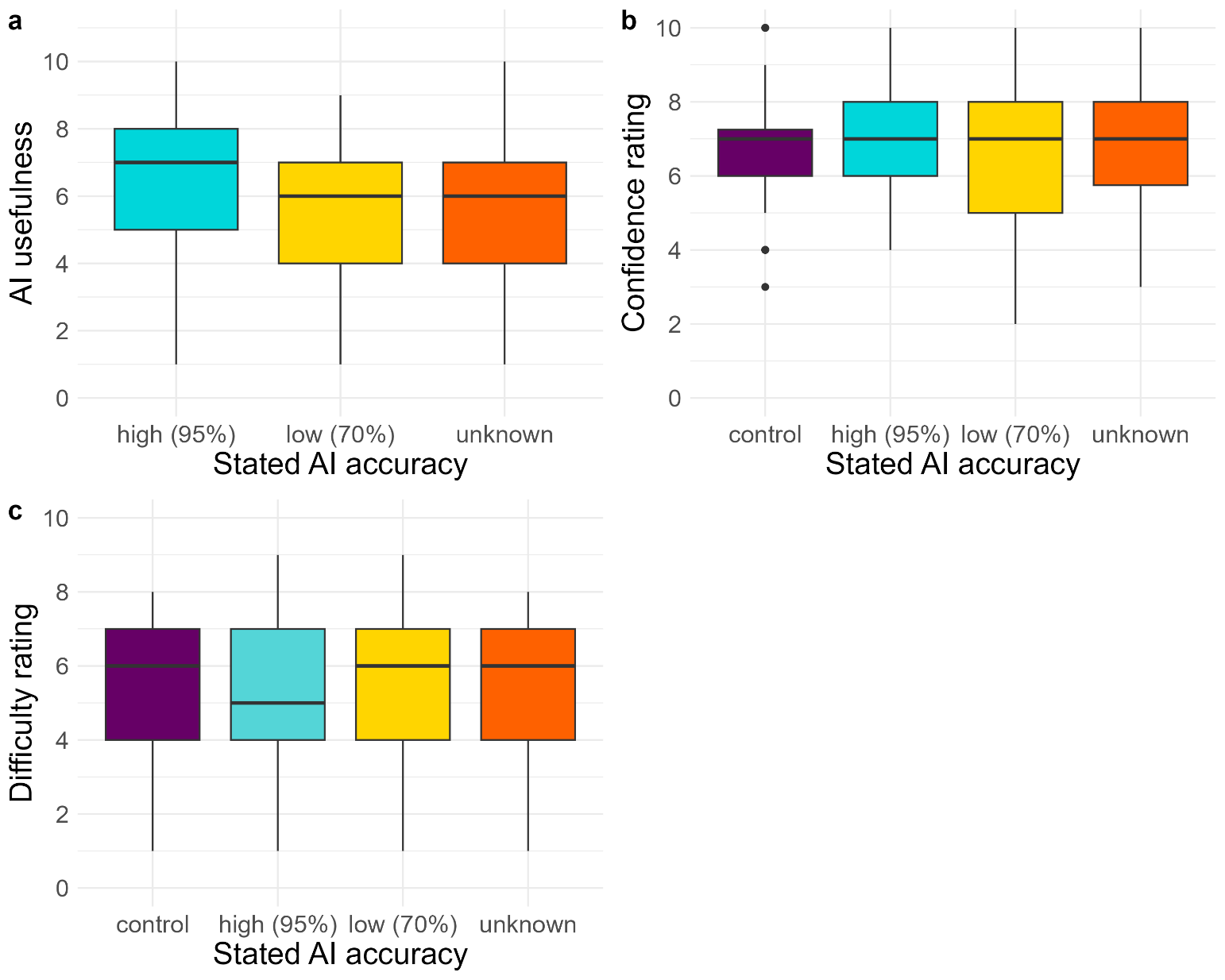
**

**Figure S1:** Participants’ ratings in each condition for (a) AI usefulness (b) confidence in decisions (c) task difficulty (Experiment 1)

|  | **Confidence** | | | | | **Difficulty** | | | | |
| --- | --- | --- | --- | --- | --- | --- | --- | --- | --- | --- |
| *Predictors* | *Estimates* | *SE* | *95% CI* | *Statistic* | *p* | *Estimates* | *SE* | *95% CI* | *Statistic* | *p* |
| Intercept | 6.65 | 0.16 | 6.34 – 6.97 | 41.81 | **<0.001** | 5.36 | 0.20 | 4.97 – 5.75 | 27.14 | **<0.001** |
| Condition [95%] | 0.15 | 0.14 | -0.12 – 0.43 | 1.09 | 0.278 | -0.14 | 0.18 | -0.49 – 0.21 | -0.80 | 0.427 |
| Condition [70%] | -0.11 | 0.14 | -0.38 – 0.17 | -0.78 | 0.438 | 0.30 | 0.18 | -0.05 – 0.65 | 1.71 | 0.088 |
| Condition [Unknown] | -0.03 | 0.14 | -0.31 – 0.24 | -0.23 | 0.816 | -0.09 | 0.18 | -0.44 – 0.26 | -0.49 | 0.625 |
| **Random Effects** | | | | | | | | | | |
| σ^2^ | 0.90 | | | | | 1.45 | | | | |
| τ_00_ | 1.43 _participant_ | | | | | 2.13 _participant_ | | | | |
| ICC | 0.61 | | | | | 0.60 | | | | |
| N | 92 _participant_ | | | | | 92 _participant_ | | | | |
| Observations | 368 | | | | | 368 | | | | |
| Marginal R^2^ / Conditional R^2^ | 0.004 / 0.615 | | | | | 0.008 / 0.599 | | | | |

**Table S3**: Results of the mixed-effects linear regression for confidence and difficulty ratings (Experiment 1).

*Notes: The fit of the model including ‘stated AI accuracy’ was not significantly better than the null model for confidence rating (χ²(3) = 3.69, p = 0.30) or difficulty rating (χ²(3) = 7.50, p = 0.06).*

#### ANOVAs for Performance

We additionally conducted ANOVAs for the performance measure as per our initial pre-registration for this experiment. To assess the effect of experimental condition on participants’ performance, a one-way, within-subject ANOVA was conducted, which revealed a significant effect of experimental condition, *F*(2.53, 235.40) = 3.29, *p* = .028, η_p_^2^ = .03. Pairwise comparisons showed that accuracy was significantly higher on the ‘unknown AI accuracy’ condition compared to the control condition where no AI advice was presented (*p* = .02). Performance on all other conditions did not differ significantly from each other.

For the next analysis, only the data from the three blocks where AI advice was presented, was included. A 2 (AI advice accuracy: accurate vs. inaccurate) x 3 (experimental condition: 95% vs. 70% vs. unknown AI accuracy) within-subject ANOVA was conducted for performance, which showed a main effect of AI advice accuracy, *F*(1, 93) = 245.43, *p* < .001, η_p_^2^ = .73, with higher accuracy observed on trials where accurate advice was presented. Neither the main effect of experimental condition, nor the interaction effect were significant (all *F*s <1.39 and *p*s > .251).

A one-way, within-subject ANOVA was conducted for AI usefulness, confidence, and difficulty ratings variables, which revealed a significant effect of experimental condition on AI usefulness, *F*(1.86, 169.45) = 10.83, *p* < .001, η_p_^2^ = .11. Pairwise comparisons indicated that participants rated usefulness of AI advice significantly higher on the ‘high stated accuracy’ condition, compared to the ‘low’ and ‘unknown’ conditions (both *p*s <.001). For confidence and difficulty ratings, the ANOVAs were not significant (all *F*s < 2.51 and *p*s > .05).

|  | **AI agreement** | | | | |
| --- | --- | --- | --- | --- | --- |
| *Predictors* | *Odds Ratios* | *SE* | *95% CI* | *Statistic* | *p* |
| Intercept | 18.50 | 2.24 | 14.59 – 23.45 | 24.11 | **<0.001** |
| Condition [Unknown] | 0.95 | 0.08 | 0.81 – 1.12 | -0.60 | 0.548 |
| Condition [70%] | 0.83 | 0.07 | 0.71 – 0.97 | -2.28 | **0.022** |
| AI advice [inaccurate] | 0.02 | 0.01 | 0.01 – 0.04 | -10.76 | **<0.001** |
| Condition [Unknown] x AI advice [inaccurate] | 0.67 | 0.16 | 0.42 – 1.07 | -1.69 | 0.092 |
| Condition [70%] x AI advice [inaccurate] | 0.82 | 0.19 | 0.52 – 1.29 | -0.86 | 0.387 |
| **Random Effects** | | | | | |
| σ^2^ | 3.29 | | | | |
| τ_00_ _TrialID_ | 1.31 | | | | |
| τ_00_ _participant_ | 0.27 | | | | |
| ICC | 0.33 | | | | |
| N _TrialID_ | 192 | | | | |
| N _participant_ | 94 | | | | |
| Observations | 13536 | | | | |
| Marginal R^2^ / Conditional R^2^ | 0.187 / 0.452 | | | | |

**Table S4**: Results of the mixed-effects logistic regression for participants’ agreement with AI predictions (Experiment 1)

*Notes: The fit of the model was significantly better compared to the null model and the ‘stated AI accuracy condition’ only model when ‘stated AI accuracy condition’ and ‘AI prediction accuracy’ were included (χ²*(3) = 108.69, *p* < .001)*.*

#### Signal Detection

Next, we examined the effects of the experimental condition and AI prediction accuracy within the framework of SDT. Sensitivity and criterion are plotted in Figure S2. Two one-way ANOVAs with experimental condition as a within-subjects factor revealed that neither the effect for sensitivity (*F*(2.61, 242.78) = 2.54, *p* = .065, η_p_^2^ = .03) nor criterion (*F*(2.88, 267.61) = 2.14, *p* = .099, η_p_^2^ = .02) was significant. The experimental conditions did not improve discriminability or alter the response strategy.

We then examined only the conditions with AI predictions and conducted two 2x3 within-subject ANOVAs with AI prediction accuracy and experimental condition as independent variables. For sensitivity, there was a main effect of AI prediction accuracy (*F*(1, 93) = 1889.45, *p* < .001, η_p_^2^ = .95), such that receiving *accurate* AI predictions improved discriminability compared to *inaccurate* predictions. The main effect for condition (*F*(1.99, 185.48) = 2.23, *p* = .111, η_p_^2^ = .02) and the interaction (*F*(1.90, 176.82) = 1.02, *p* = .360, η_p_^2^ = .01) were non-significant. For criterion, the main effect of condition was not significant (*F*(1.88, 174.82) = 1.70, *p* = .187, η_p_^2^ = .02), but the main effect for AI prediction accuracy (*F*(1, 93) = 4.72, *p* = .032, η_p_^2^ = .05) and the interaction were significant (*F*(1.96, 181.96) = 3.35, *p* = .038, η_p_^2^ = .03). Pairwise comparisons for the interaction showed that, when AI predictions were *accurate*, there was a more liberal response bias in the *unknown* condition compared to the *high condition* (*p* = .046) i.e. participants were more likely to say *match*. Additionally, the response bias was more liberal when predictions were *inaccurate* in the *high* (*p* = .016) and *low* conditions (*p* = .03).

Overall, *accurate* AI predictions improved participants’ sensitivity compared to *inaccurate* predictions, but this did not improve overall sensitivity in the conditions with AI predictions. Although sensitivity appears to be higher in the conditions with AI predictions (and of these, highest in the *unknown* condition), these were not significantly different from the *control c*ondition. Therefore, participants sensitivity did not change as a result of the experimental condition, but did change their criterion, especially when the predictions were *accurate*.


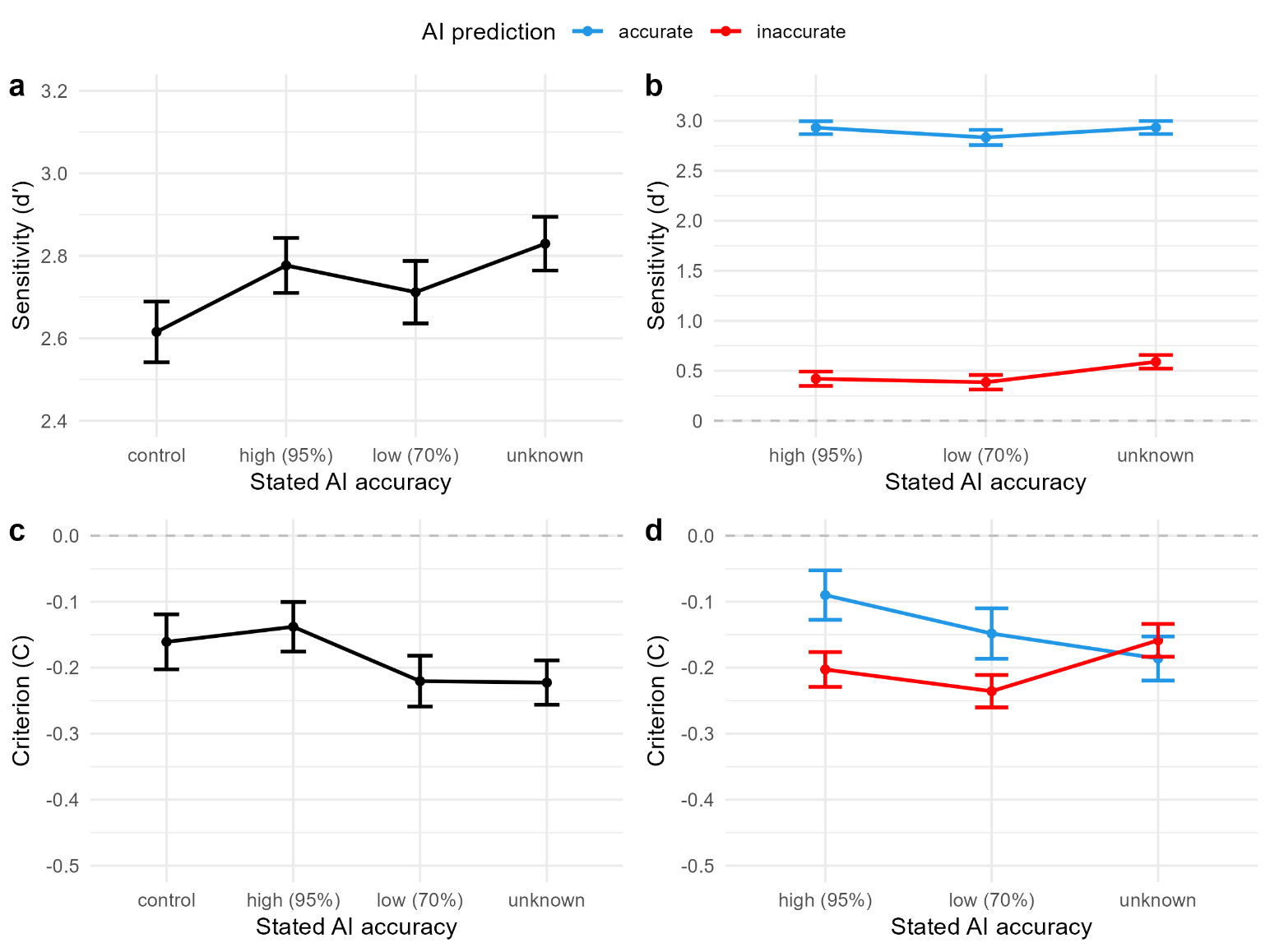


**Figure S2**: (a) Sensitivity d’ in all four conditions; (b) Sensitivity d’ in the three conditions with AI predictions by accuracy of AI predictions; (c) Criterion C in all four conditions; (d) Criterion C in the three conditions with AI predictions by accuracy of AI predictions.

# Experiment 2

### Results

### AI usefulness, confidence, and difficulty

Again, we were interested in participants’ perceptions of AI usefulness, confidence in their decisions, and task difficulty (Figure S3). Three mixed-effects linear regressions were conducted for these variables, which were regressed on mismatch frequency, with participants as a random factor (see Table S5). No effect of mismatch frequency was observed on AI usefulness, confidence or difficulty ratings.


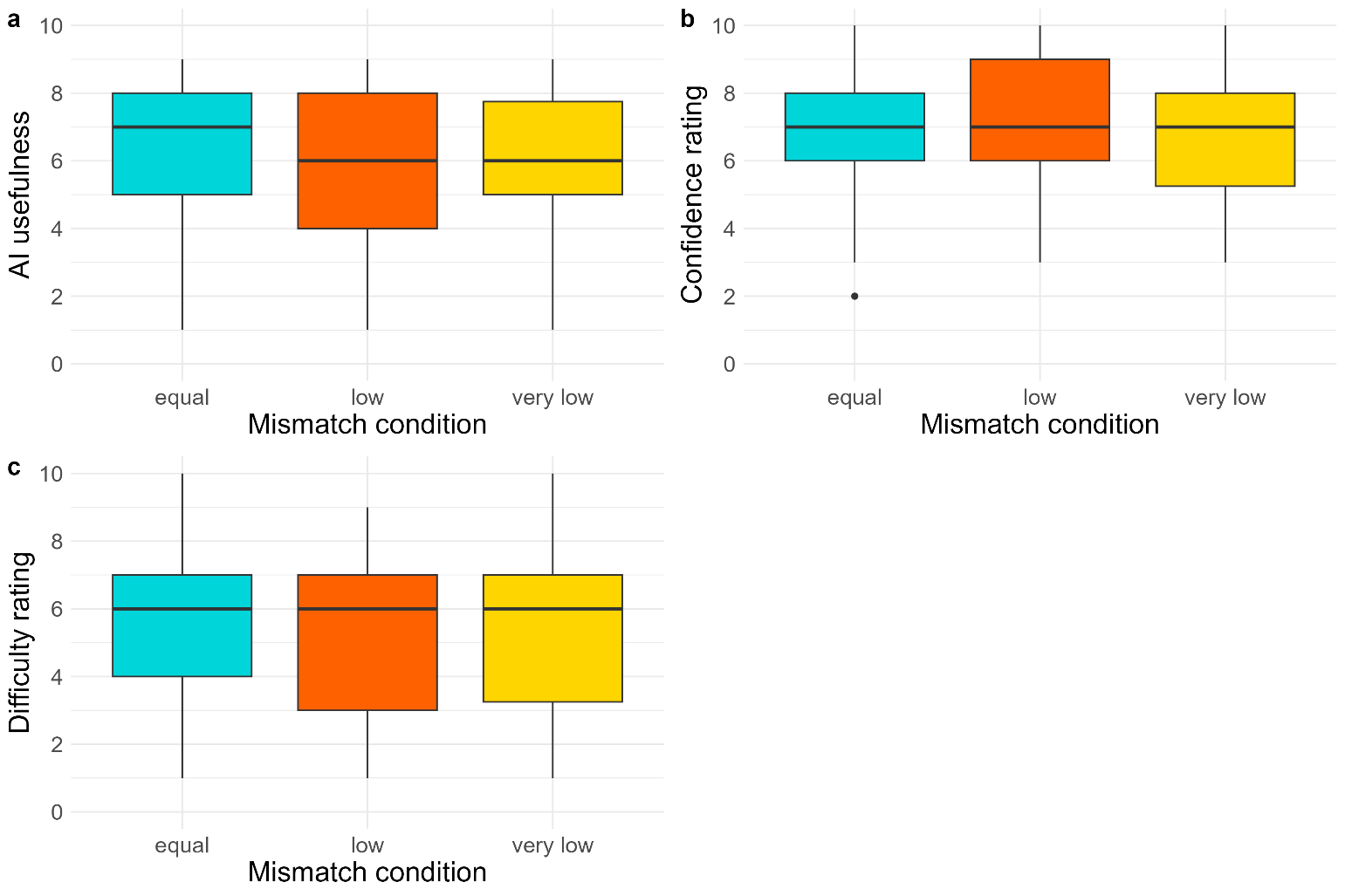


**Figure S3**: Participants’ ratings in each condition for (a) AI usefulness (b) confidence in decisions (c) task difficulty (Experiment 2)

|  | **AI usefulness** | | | | | **Confidence** | | | | | **Difficulty** | | | | |
| --- | --- | --- | --- | --- | --- | --- | --- | --- | --- | --- | --- | --- | --- | --- | --- |
| *Predictors* | *Estimates* | *SE* | *95% CI* | *Statistic* | *p* | *Estimates* | *SE* | *95% CI* | *Statistic* | *p* | *Estimates* | *SE* | *95% CI* | *Statistic* | *p* |
| Intercept | 6.14 | 0.24 | 5.66 – 6.62 | 25.36 | **<0.001** | 7.04 | 0.21 | 6.63 – 7.45 | 33.61 | **<0.001** | 5.60 | 0.25 | 5.11 – 6.10 | 22.24 | **<0.001** |
| Condition [low] | -0.13 | 0.22 | -0.56 – 0.31 | -0.58 | 0.561 | 0.05 | 0.16 | -0.27 – 0.37 | 0.31 | 0.753 | -0.27 | 0.20 | -0.67 – 0.13 | -1.34 | 0.183 |
| Condition [very low] | -0.19 | 0.22 | -0.63 – 0.24 | -0.87 | 0.383 | -0.08 | 0.16 | -0.40 – 0.24 | -0.47 | 0.637 | -0.28 | 0.20 | -0.68 – 0.12 | -1.40 | 0.163 |
| **Random Effects** | | | | | | | | | | | | | | | |
| σ^2^ | 1.89 | | | | | 1.03 | | | | | 1.58 | | | | |
| τ_00_ | 2.68 _participant_ | | | | | 2.39 _participant_ | | | | | 3.36 _participant_ | | | | |
| ICC | 0.59 | | | | | 0.70 | | | | | 0.68 | | | | |
| N | 78 _participant_ | | | | | 78 _participant_ | | | | | 78 _participant_ | | | | |
| Observations | 234 | | | | | 234 | | | | | 234 | | | | |
| Marginal R^2^ / Conditional R^2^ | 0.001 / 0.587 | | | | | 0.001 / 0.698 | | | | | 0.003 / 0.681 | | | | |

**Table S5**: Results of the mixed-effects linear regression for AI usefulness, confidence, and difficulty ratings (Experiment 2).

*Notes: Including ‘mismatch frequency’ did not significantly improve model fit over the null model for AI usefulness (χ²*(2) = 0.80, *p* = .671*), confidence (χ²*(2) = 0.63, *p* = .728*), or difficulty (χ²*(2) = 2.51, *p* = .285*).*

|  | **AI agreement** | | | | |
| --- | --- | --- | --- | --- | --- |
| *Predictors* | *Odds Ratios* | *SE* | *95% CI* | *Statistic* | *p* |
| Intercept | 11.61 | 2.39 | 7.75 – 17.38 | 11.90 | **<0.001** |
| Condition [low] | 1.30 | 0.34 | 0.77 – 2.17 | 0.99 | 0.324 |
| Condition [very low] | 1.08 | 0.28 | 0.64 – 1.80 | 0.28 | 0.779 |
| AI advice [inaccurate] | 0.04 | 0.02 | 0.01 – 0.13 | -5.04 | **<0.001** |
| Condition [low] x AI advice [inaccurate] | 1.06 | 0.99 | 0.17 – 6.63 | 0.06 | 0.953 |
| Condition [very low] x AI advice [inaccurate] | 0.69 | 0.64 | 0.11 – 4.23 | -0.40 | 0.687 |
| **Random Effects** | | | | | |
| σ^2^ | 3.29 | | | | |
| τ_00_ _TrialID_ | 1.09 | | | | |
| τ_00_ _participant_ | 0.61 | | | | |
| ICC | 0.34 | | | | |
| N _TrialID_ | 120 | | | | |
| N _participant_ | 78 | | | | |
| Observations | 9360 | | | | |
| Marginal R^2^ / Conditional R^2^ | 0.144 / 0.435 | | | | |

**Table S6**: Results of the mixed-effects logistic regression for participants’ agreement with AI predictions (Experiment 2)

*Notes: The fit of the model was significantly better compared to the null model and the ‘mismatch frequency’ only model when ‘mismatch frequency’ and ‘AI prediction accuracy’ were included (χ²*(3) = 61.18, *p* < .001)*.*

#### Signal Detection

The sensitivity and criterion scores are presented in Figure S4. A 2x3 within-subject ANOVA revealed a main effect of condition (*F*(1.66, 127.70) = 36.71, *p* < .001, η_p_^2^ = .32), wherein sensitivity was lower in the *very low* condition compared to the other two conditions (both *p*s < .001). A main effect of AI prediction accuracy (*F*(1, 77) = 865.83, *p* < .001, η_p_^2^ = .92), and a significant interaction (*F*(1.83, 140.98) = 179.30, *p* < .001, η_p_^2^ = .70) on sensitivity were also observed. Pairwise comparisons indicated that, for *accurate* predictions, sensitivity was significantly higher in the *low* condition compared to the *very low* condition, and for the *equal* condition compared to both, *low* and *very low,* conditions (all *p*s < .001). For *inaccurate* predictions, sensitivity was significantly lower in the *equal* condition compared to *low* and *very low* conditions (both *p*s < .001), and sensitivity was lower in the *very low* condition compared to the *low* condition (*p* = .032). Sensitivity was significantly higher for the *accurate* compared to *inaccurate* prediction trials across all three conditions (all *p*s < .001).

Further, there was a main effect of condition (*F*(1.77, 136.05) = 94.75, *p* < .001, η_p_^2^ = .55) and AI prediction accuracy (*F*(1, 77) = 65.25, *p* < .001, η_p_^2^ = .46), and a significant interaction (*F*(1.98, 152.43) = 38.19, *p* < .001, η_p_^2^ = .33) for criterion. Criterion was significantly lower for the *accurate* compared to *inaccurate* prediction trials in the *low* and *very low* conditions (both *p*s < .001), but not in the *equal* condition (*p* = .239). For *accurate* prediction trials, criterion was significantly higher in the *equal* condition compared to the other two conditions, and in the *low* compared to *very low* condition (all *p*s < .001). For *inaccurate* prediction trials, criterion was higher in the *equal* condition compared to others (both *p*s < .001), and in the *very low* condition compared to the *low* condition (*p* = .032). It is worth noting here that while the corrections for hit and false alarm rates of 0 and 1 allow us to calculate sensitivity and criterion values, they are based on very small samples in the inaccurate prediction cases, and should be interpreted with care. Participants showed a relatively low response bias in the *equal* condition for *accurate* and *inaccurate* AI predictions, as reflected in criterion values near zero. With lower mismatch trials, participants exhibited an increased response bias such that they were more likely to respond *match*. These findings are in line with Papesh and Goldinger (2014), who also reported a higher response bias in the low mismatch frequency condition (note that the signs are reversed for the finding from Papesh and Goldinger as they classified correct identification of mismatches as *hits*, whereas we classify correct identification of matches as *hits*).


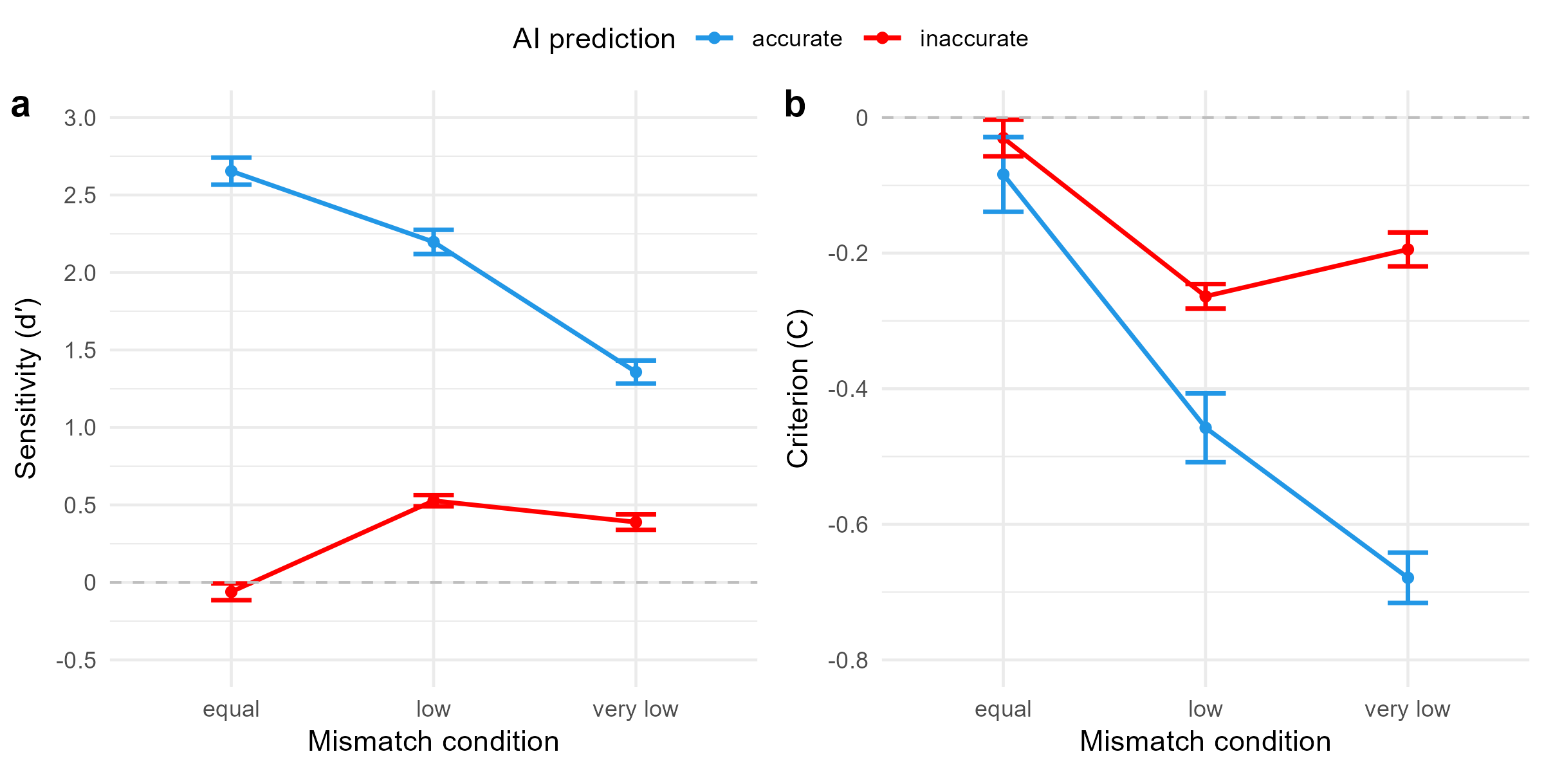


**Figure S4**: Sensitivity d’ (a) and Criterion C (b) in the three mismatch conditions by accuracy of AI predictions.

#### Additional analyses

In order to evaluate whether there may have been statistical learning involved while completing each block, we divided the trials within each block into two halves based on the order of completion of the trials. The first 20 trials completed by each participant in each block were classified as the *first half* and the rest as the *second half*. We then conducted a logistic mixed-effects regression with *block half* and *mismatch condition* as fixed factors, and participants and face pairs as random factors. The results of the regression analysis are presented in Table S7. The main effects were non-significant. There appears to be an interaction effect, however, the fit of the model, was not significantly better than the null model. Therefore, we did not explore the interaction further.

|  | **Performance** | | | | |
| --- | --- | --- | --- | --- | --- |
| *Predictors* | *Odds Ratios* | *SE* | *95% CI* | *Statistic* | *p* |
| Intercept | 10.46 | 2.36 | 6.72 – 16.27 | 10.40 | **<0.001** |
| Block half [second] | 1.06 | 0.12 | 0.85 – 1.33 | 0.51 | 0.612 |
| Mismatch condition [Low] | 1.49 | 0.43 | 0.84 – 2.64 | 1.38 | 0.168 |
| Mismatch condition [Very low] | 1.20 | 0.35 | 0.68 – 2.12 | 0.64 | 0.522 |
| Half [second] x Mismatch condition [Low] | 0.69 | 0.12 | 0.50 – 0.97 | -2.15 | **0.032** |
| Half [second] x Mismatch condition [Very low] | 0.83 | 0.13 | 0.60 – 1.14 | -1.17 | 0.242 |
| **Random Effects** | | | | | |
| σ^2^ | 3.29 | | | | |
| τ_00_ _TrialID_ | 1.35 | | | | |
| τ_00_ _participant_ | 0.71 | | | | |
| ICC | 0.38 | | | | |
| N _TrialID_ | 120 | | | | |
| N _participant_ | 78 | | | | |
| Observations | 9360 | | | | |
| Marginal R^2^ / Conditional R^2^ | 0.003 / 0.387 | | | | |

**Table S7**: Results of the mixed-effects logistic regression for participants’ performance on the first half of trials vs. the second half of trials in each block (Experiment 2).

*Notes: The fit of the ‘half’ only model was not significantly better than the null model (χ²(2*) *= 0.59, p = .746), nor was the fit of the model with ‘half’ and ‘mismatch frequency’ significantly better compared to the null model or the ‘half’ only model (*χ²(3) *= 7.23, p = .065).*

Further, we examined only the block that was completed first by each participant and compared the performance with the overall performance from all blocks (see Table 4) in order to evaluate whether performance was negatively influenced as a result of time spent performing the task. We conducted two logistic mixed-effects regressions (see Table S8), similar to those in Table 4, except we only included the block that was completed first by each participant. The results were identical to the original results with the whole dataset in the model with mismatch condition and AI prediction accuracy as predictors (Model 1). In the model with mismatch condition and trial type (Model 2) too, results were similar, except a significant interaction (*p* = .047), which was verging on significance in the original analysis (*p* = .053). We followed both up with pairwise comparisons of estimated marginal means using Holm correction (see Table S9). We found that, in the equal condition, the performance on the match trials compared to mismatch trials, which was significantly better with the whole dataset, was not significantly different when analysing the first blocks only. This points to a potential, albeit weak, carry over effect of the liberal response bias when the equal condition is preceded by one of the low mismatch conditions. However, considering that other comparisons were not different when comparing the whole dataset and first block, we cannot conclude that there was a response bias as a result of time.

|  | | **Performance (Model 1)** | | | | | | **Performance (Model 2)** | | | | |
| --- | --- | --- | --- | --- | --- | --- | --- | --- | --- | --- | --- | --- |
| *Predictors* | | *Odds Ratios* | | *SE* | *95% CI* | *Statistic* | *p* | *Odds Ratios* | *SE* | *95% CI* | *Statistic* | *p* |
| Intercept | | 10.26 | | 2.86 | 5.95 – 17.72 | 8.36 | **<0.001** | 12.94 | 4.36 | 6.69 – 25.03 | 7.61 | **<0.001** |
| Mismatch condition [Low] | | 1.33 | | 0.53 | 0.61 – 2.88 | 0.72 | 0.473 | 1.16 | 0.51 | 0.49 – 2.74 | 0.33 | 0.741 |
| Mismatch condition [Very low] | | 0.95 | | 0.37 | 0.45 – 2.03 | -0.13 | 0.900 | 0.67 | 0.29 | 0.29 – 1.54 | -0.95 | 0.344 |
| AI prediction [inaccurate] | | 0.21 | | 0.14 | 0.05 – 0.78 | -2.32 | **0.020** |  |  |  |  |  |
| Mismatch condition [Low] x AI prediction [inaccurate] |  | | 0.76 | 0.73 | 0.11 – 4.98 | -0.29 | 0.771 |  |  |  |  |  |
| Mismatch condition [Very low] x AI prediction [inaccurate] | | 1.92 | | 1.82 | 0.30 – 12.29 | 0.69 | 0.492 |  |  |  |  |  |
| trial type [mismatch] | |  | |  |  |  |  | 0.50 | 0.19 | 0.24 – 1.04 | -1.86 | 0.062 |
| Mismatch condition [Low] x trial type [mismatch] | |  | |  |  |  |  | 0.25 | 0.17 | 0.06 – 0.98 | -1.98 | **0.047** |
| Mismatch condition [Very low] x trial type [mismatch] | |  | |  |  |  |  | 5.63 | 5.56 | 0.81 – 38.97 | 1.75 | 0.080 |
| **Random Effects** | | | | | | | | | | | | |
| σ^2^ | | 3.29 | | | | | | 3.29 | | | | |
| τ_00_ | | 0.98 _TrialID_ | | | | | | 0.97 _TrialID_ | | | | |
|  | | 0.93 _participant_ | | | | | | 0.93 _participant_ | | | | |
| ICC | | 0.37 | | | | | | 0.37 | | | | |
| N | | 120 _TrialID_ | | | | | | 120 _TrialID_ | | | | |
|  | | 78 _participant_ | | | | | | 78 _participant_ | | | | |
| Observations | | 3120 | | | | | | 3120 | | | | |
| Marginal R^2^ / Conditional R^2^ | | 0.031 / 0.387 | | | | | | 0.037 / 0.390 | | | | |

**Table S8**: Results of the mixed-effects logistic regression for participants’ performance on the first block completed only (Experiment 2).

*Notes: The fit of model 1, which included ‘mismatch condition’ and ‘AI prediction accuracy’ was significantly better than the ‘mismatch condition’ only and the null model (χ²(3*) *= 13.58, p = .004). The fit of the model 2, which included ‘mismatch condition’ and ‘trial type’ was significantly better than the ‘mismatch condition’ only and the null model (*χ²(3) *= 16.04, p = .001).*

|  | *Whole dataset* | | | *First block only* | | |
| --- | --- | --- | --- | --- | --- | --- |
|  | Estimate | SE | *p* | Estimate | SE | *p* |
| **Equal** |  |  |  |  |  |  |
| Match-Mismatch | 0.884 | 0.36 | **0.014** | 0.695 | 0.37 | 0.062 |
| **Low** |  |  |  |  |  |  |
| Match-Mismatch | 2.212 | 0.59 | **<.001** | 2.08 | 0.59 | **<.001** |
| **Very Low** |  |  |  |  |  |  |
| Match-Mismatch | 0.529 | 0.81 | 0.516 | -1.034 | 0.91 | 0.258 |

|  | *Whole dataset* | | | *First block only* | | |
| --- | --- | --- | --- | --- | --- | --- |
|  | Estimate | SE | *p* | Estimate | SE | *p* |
| **Match** |  |  |  |  |  |  |
| Equal-Low | 0.003 | 0.32 | 0.99 | -0.145 | 0.44 | 0.742 |
| Equal-Very low | 0.344 | 0.32 | 0.603 | 0.405 | 0.43 | 0.687 |
| Low-Very low | 0.34 | 0.27 | 0.603 | 0.55 | 0.39 | 0.474 |
| **Mismatch** |  |  |  |  |  |  |
| Equal-Low | 1.332 | 0.61 | 0.084 | 1.24 | 0.67 | 0.127 |
| Equal-Very low | -0.011 | 0.83 | 0.99 | -1.324 | 0.97 | 0.173 |
| Low-Very low | -1.343 | 0.97 | 0.329 | -2.564 | 1.09 | 0.055 |

**Table S9**: Pairwise comparisons of estimated marginal means of the interaction in the logistic mixed-effects models in Table S8 and Table 4.

**Experiment 3**

## Results

### AI usefulness, confidence, and task difficulty

Participants’ AI usefulness, confidence, and difficulty ratings are provided in Figure S5. Three mixed-effects linear regression analyses were conducted for each variable. Each variable was regressed on the advice condition as a fixed factor, with participants as a random factor (see Table S11). The analysis indicated that advice condition did not significantly affect any of the three variables.


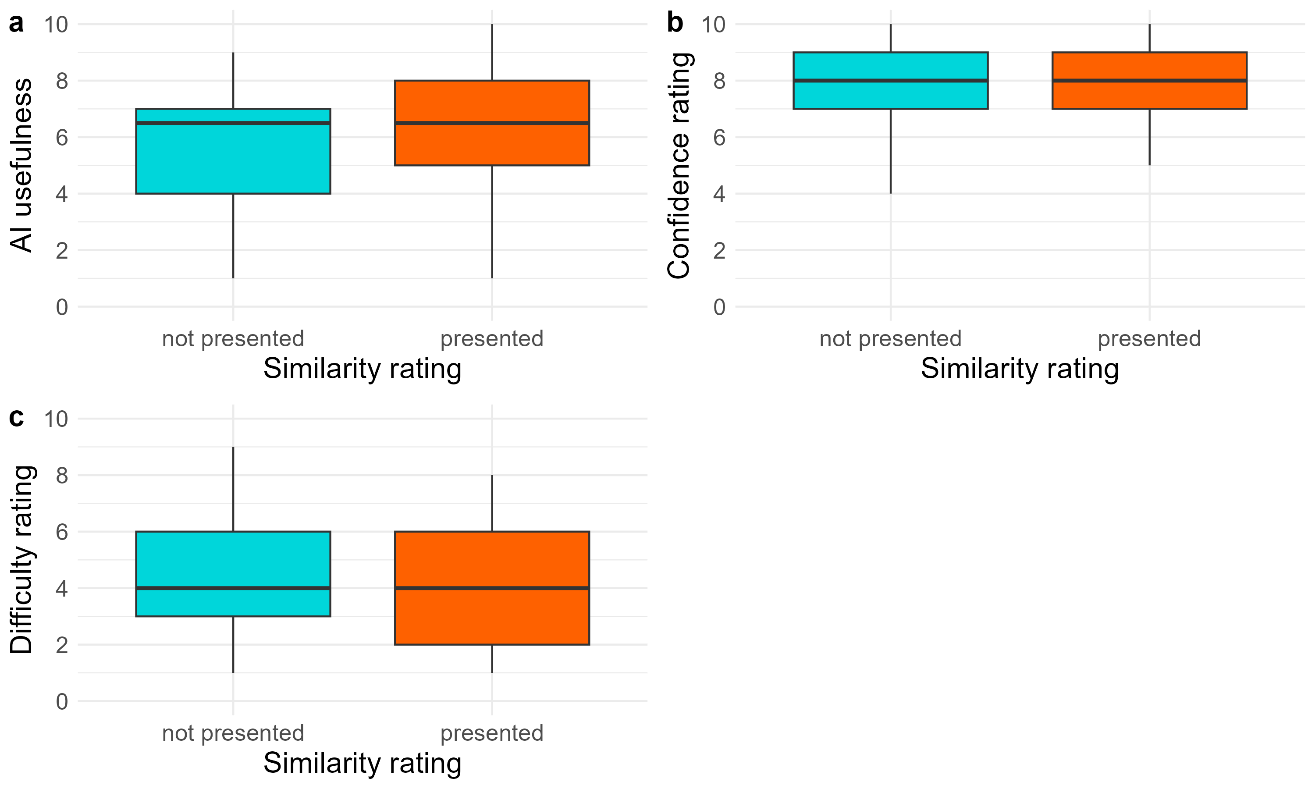


**Figure S5:** Participants’ ratings in each condition for (a) AI usefulness (b) confidence in decisions (c) task difficulty (Experiment 3).

|  | **AI Agreement** | | | | |
| --- | --- | --- | --- | --- | --- |
| *Predictors* | *Odds Ratios* | *SE* | *95% CI* | *Statistic* | *p* |
| Intercept | 27.16 | 4.45 | 19.70 – 37.45 | 20.15 | **<0.001** |
| Similarity ratings [presented] | 1.31 | 0.10 | 1.13 – 1.53 | 3.49 | **<0.001** |
| AI advice [inaccurate] | 0.02 | 0.01 | 0.01 – 0.04 | -8.72 | **<0.001** |
| Similarity ratings [presented] x AI advice [inaccurate] | 1.05 | 0.21 | 0.70 – 1.55 | 0.23 | 0.820 |
| **Random Effects** | | | | | |
| σ^2^ | 3.29 | | | | |
| τ_00_ _TrialID_ | 2.23 | | | | |
| τ_00_ _participant_ | 0.47 | | | | |
| ICC | 0.45 | | | | |
| N _TrialID_ | 160 | | | | |
| N _participant_ | 75 | | | | |
| Observations | 12000 | | | | |
| Marginal R^2^ / Conditional R^2^ | 0.167 / 0.543 | | | | |

**Table S10**: Results of the mixed-effects logistic regression for participants’ agreement with AI predictions (Experiment 3).

*Notes: The model fit showed a significant improvement over the null model and the ‘similarity ratings’ only model when ‘similarity ratings’ and ‘AI prediction accuracy’ were included (χ²*(3) = 76.09, *p* < .001*).*

|  | | | **AI usefulness** | | | | **Confidence** | | | | | **Difficulty** | | | | |
| --- | --- | --- | --- | --- | --- | --- | --- | --- | --- | --- | --- | --- | --- | --- | --- | --- |
| *Predictors* | *Estimates* | *SE* | | *95% CI* | *Statistic* | *p* | *Estimates* | *SE* | *95% CI* | *Statistic* | *p* | *Estimates* | *SE* | *95% CI* | *Statistic* | *p* |
| Intercept | 5.83 | 0.26 | | 5.32 – 6.34 | 22.45 | **<0.001** | 7.77 | 0.15 | 7.47 – 8.07 | 50.70 | **<0.001** | 4.39 | 0.26 | 3.86 – 4.91 | 16.61 | **<0.001** |
| Similarity ratings [presented] | 0.19 | 0.19 | | -0.19 – 0.56 | 0.98 | 0.328 | 0.16 | 0.11 | -0.06 – 0.38 | 1.42 | 0.158 | -0.31 | 0.19 | -0.69 – 0.06 | -1.67 | 0.097 |
| **Random Effects** | | | | | | | | | | | | | | | | |
| σ^2^ | | | 1.25 | | | | 0.43 | | | | | 1.24 | | | | |
| τ_00_ | | | 3.47 _participant_ | | | | 1.22 _participant_ | | | | | 3.64 _participant_ | | | | |
| ICC | | | 0.73 | | | | 0.74 | | | | | 0.75 | | | | |
| N | | | 70 _participant_ | | | | 70 _participant_ | | | | | 70 _participant_ | | | | |
| Observations | | | 140 | | | | 140 | | | | | 140 | | | | |
| Marginal R^2^ / Conditional R^2^ | | | 0.002 / 0.735 | | | | 0.004 / 0.740 | | | | | 0.005 / 0.747 | | | | |

**Table S11:** Results of the mixed-effects linear regression for AI usefulness, confidence, and difficulty ratings (Experiment 3).

*Notes: Including ‘similarity ratings’ did not significantly improve model fit over the null model for AI usefulness (χ²*(1) = 0.97, *p* = .324*), confidence (χ²*(1) = 2.01, *p* = .156*), or difficulty (χ²*(1) = 2.77, *p* = .096*).*

#### Signal Detection

The means and SE for sensitivity and criterion are provided in Figure S6. A 2x2 within-subjects ANOVA revealed no significant effect of similarity condition (*F*(1, 74) = 0.02, *p* = .899, η_p_^2^ < .001), but a main effect of AI prediction accuracy (*F*(1, 74) = 703.92, *p* < .001, η_p_^2^ = .90) and a significant interaction (*F*(1, 74) = 5.53, *p* = .021, η_p_^2^ = .07) were found for sensitivity. Bonferroni adjusted pairwise comparisons showed that, when AI predictions were *accurate*, sensitivity was significantly higher when *similarity ratings* were presented (*p* = .026), but a similar effect was not observed for *inaccurate* predictions (*p* = .366). For criterion, a 2x2 within-subject ANOVA revealed no main effect of similarity condition (*F*(1, 74) = 0.47, *p* = .495, η_p_^2^ = .006) or an interaction (*F*(1, 74) = 0.16, *p* = .687, η_p_^2^ = .002). A significant main effect of AI prediction accuracy was observed (*F*(1, 74) = 62.10, *p* < .001, η_p_^2^ = .46), such that, participants showed a greater liberal response bias when *inaccurate* AI predictions were presented. Together, these findings indicate that similarity ratings improved discriminability when predictions were *accurate*, and when *inaccurate*, participants may have defaulted to responding *match*, irrespective of whether similarity ratings were presented.


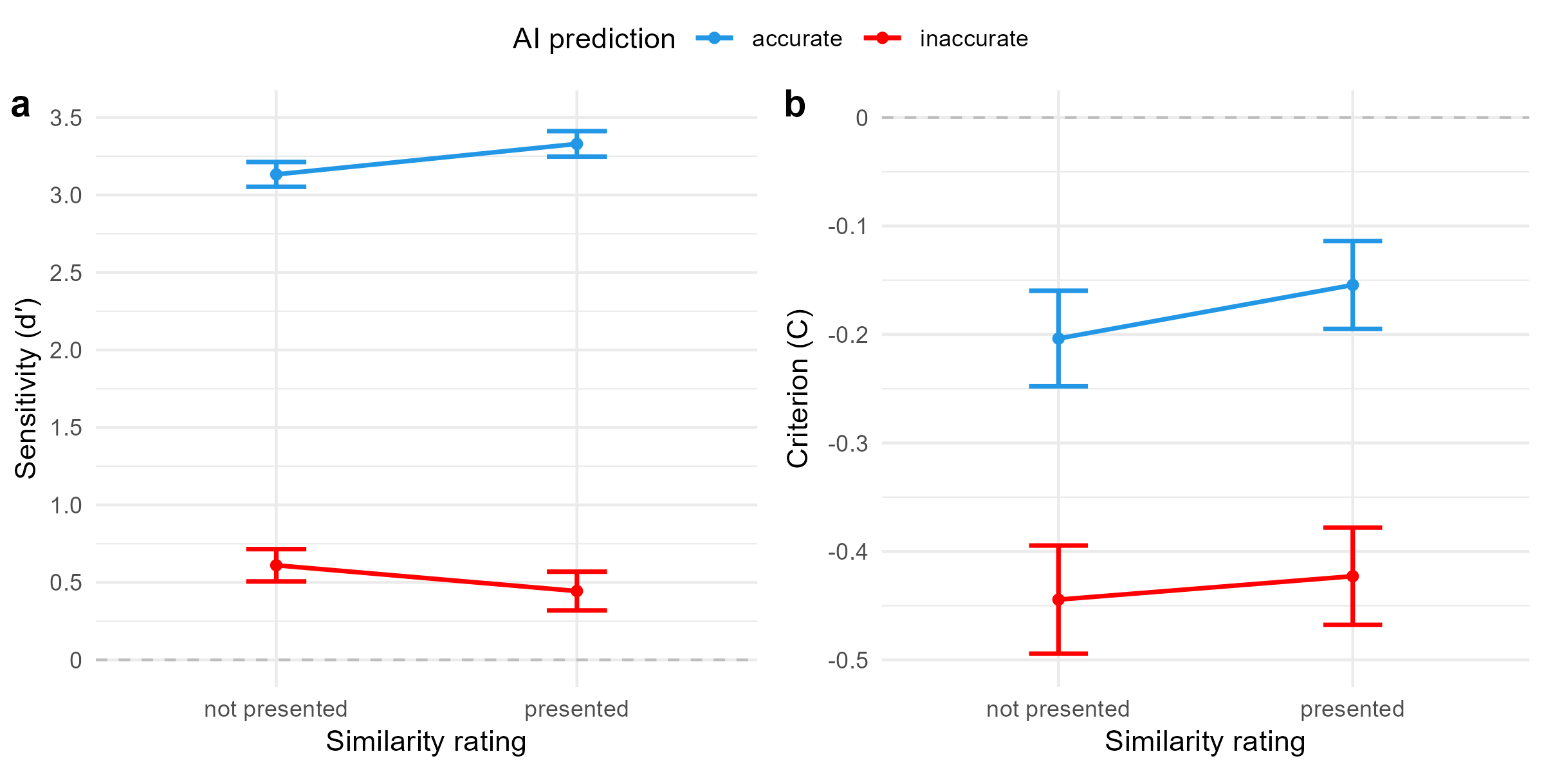


**Figure S6**: Sensitivity d’ (a) and Criterion C (b) in the similarity ratings conditions by accuracy of AI predictions.

# References

Stanislaw, H., & Todorov, N. (1999). Calculation of signal detection theory measures. *Behavior research methods, instruments, & computers*, *31*(1), 137-149. https://doi.org/10.3758/BF03207704
